# Insight into different environmental niches adaptation and allergenicity from the *Cladosporium sphaerospermum* genome, a common human allergy-eliciting Dothideomycetes

**DOI:** 10.1038/srep27008

**Published:** 2016-05-31

**Authors:** Su Mei Yew, Chai Ling Chan, Yun Fong Ngeow, Yue Fen Toh, Shiang Ling Na, Kok Wei Lee, Chee-Choong Hoh, Wai-Yan Yee, Kee Peng Ng, Chee Sian Kuan

**Affiliations:** 1 Department of Medical Microbiology, Faculty of Medicine, University of Malaya, 50603, Kuala Lumpur, Malaysia; 2 Department of Pre-Clinical Sciences, Faculty of Medicine and Health Sciences, Universiti Tunku Abdul Rahman, Bandar Sungai Long, 43000 Kajang, Selangor Darul Ehsan, Malaysia; 3 Codon Genomics SB, No. 26, Jalan Dutamas 7, Taman Dutamas, Balakong, 43200 Seri Kembangan, Selangor Darul Ehsan, Malaysia

## Abstract

*Cladosporium sphaerospermum*, a dematiaceous saprophytic fungus commonly found in diverse environments, has been reported to cause allergy and other occasional diseases in humans. However, its basic biology and genetic information are largely unexplored. A clinical isolate *C. sphaerospermum* genome, UM 843, was re-sequenced and combined with previously generated sequences to form a model 26.89 Mb genome containing 9,652 predicted genes. Functional annotation on predicted genes suggests the ability of this fungus to degrade carbohydrate and protein complexes. Several putative peptidases responsible for lung tissue hydrolysis were identified. These genes shared high similarity with the *Aspergillus* peptidases. The UM 843 genome encodes a wide array of proteins involved in the biosynthesis of melanin, siderophores, cladosins and survival in high salinity environment. In addition, a total of 28 genes were predicted to be associated with allergy. Orthologous gene analysis together with 22 other Dothideomycetes showed genes uniquely present in UM 843 that encode four class 1 hydrophobins which may be allergens specific to *Cladosporium*. The mRNA of these hydrophobins were detected by RT-PCR. The genomic analysis of UM 843 contributes to the understanding of the biology and allergenicity of this widely-prevalent species.

*Cladosporium* species is a member of the largest group of dematiaceous hyphomycetes belonging to the order Capnodiales in the class Dothideomycetes. *Cladosporium sphaerospermum* is a member of a heterogeneous complex comprising several genetically and morphologically distinctive species^1^. It is a saprophyte found ubiquitously in natural and man-made environments such as indoor and outdoor air, soil, decaying vegetation, paint, silicone and textiles^1^. Fascinatingly, *C. sphaerospermum* is the dominant fungal species from the radiation-contaminated wall and soil at the damaged nuclear power plant in Chernobyl^2^. It has been postulated that melanin enhances the growth of the fungus treated with ionizing radiation by harnessing the energy released from radioactive elements and converting it to metabolic energy.

*C. sphaerospermum* occasionally causes phaeohyphomycosis irrespective of the host’s immune status^3^,^4^. Generally, *Cladosporium* spp. are also a strong aero-allergen causing allergic diseases of the respiratory tract and intrabronchial lesions^5^. Among the *Cladopsorium* spp., *Cladosporium herbarum* is the best studied with a total of 14 allergens identified^6^. On the other hand, there is scarce data on *C. sphaerospermum* allergens. This species is poorly described despite its potential pathogenicity and allergenicity. In our previous retrospective laboratory study^7^, it was found to be the most often isolated species and frequently recovered from blood. Here, we used strain UM 843 isolated from the previous survey^7^,^8^ as a model for genomic analysis. We re-sequenced the genome using a 5-kb insert size DNA library and combined the sequences with the previous small insert DNA library to improve the genome assembly. To our knowledge, this report is the first comprehensive *in silico* genomic characterisation of the *C. sphaerospermum* species. We addressed two main questions in this work: 1) what genomic picture underpins the adaptation of *C. sphaerospermum* survival in diverse environmental niches? 2) what are the common and specific allergens in *Cladosporium* that are potential causes of human allergies?

## Results and Discussion

### *Cladosporium sphaerospermum* UM 843 identity confirmation

**Figure 1:**
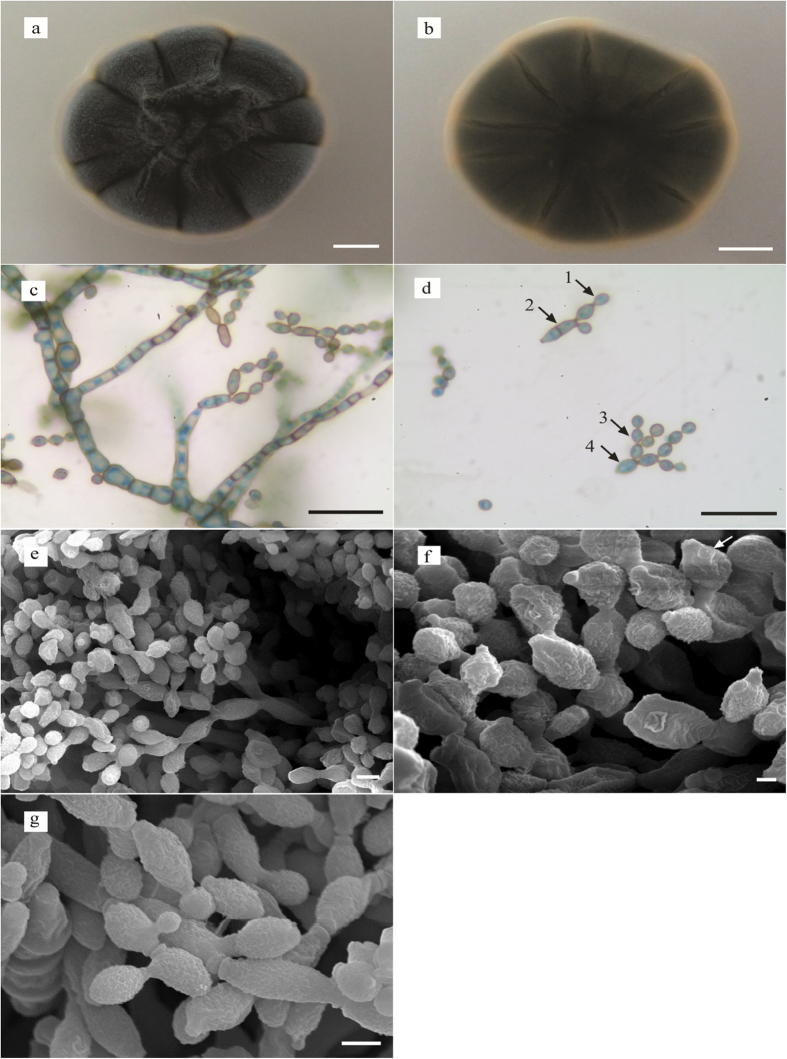
Morphology of *C. sphaerospermum* UM 843. Colonial morphology front (**a**) and reverse (**b**) of *C. sphaerospermum* UM 843 on SDA after 7-day incubation. Light micrograph showing ramoconidia (d 1 and d 3) and conidia (d 2 and d 4). ×630 magnification, bars 20 μm. Observation under scanning electron micrograph showing (**e,f,g**) conidiophores bearing conidium (**e**, ×2000 magnification, bar 3 μm), periclinal rim (**f**, ×5000 magnification bar 1 μm) and verruculose surface of conidia (**g**, ×5000 magnification, bar 2 μm).

**Figure 2:**
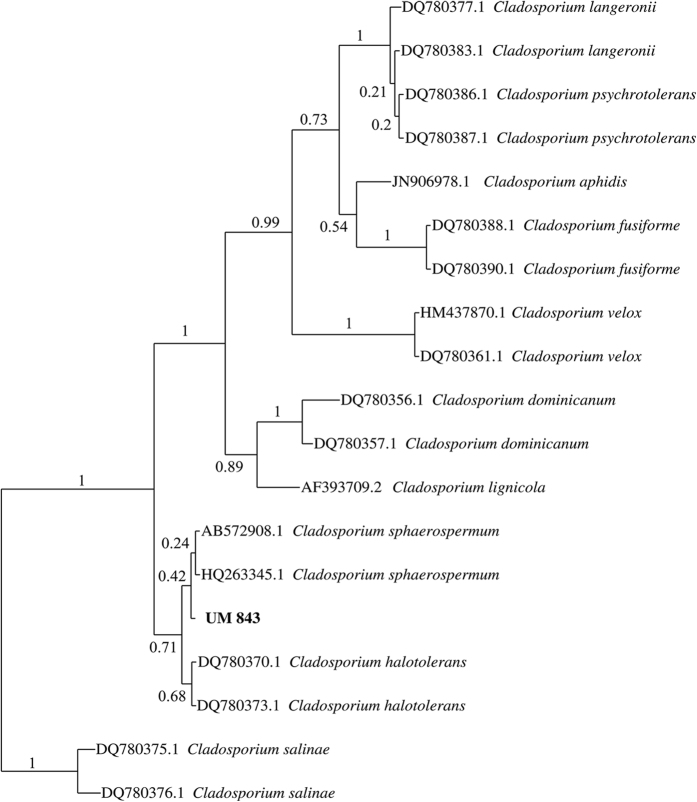
ITS-based phylogenetic tree of *C. sphaerospermum* species complex. Bayesian phylogenetic tree of *C. sphaerospermum* species complex generated using ITS1-5.8SRNA-ITS2 rDNA region, with *C. salinae* as an outgroup strain. Numbers on the nodes indicate Bayesian poterior probability based on 100 sampling frequency for a total of 150,000 generations. UM 843 was resolved as *C. sphaerospermum*.

The UM 843 colony on SDA was flat, radially furrowed with a wrinkled centre forming a crater-like structure (Fig. 1a,b). It had an olivaceous green pigment on the upper surface (Fig. 1a) and was black-green on the reverse side (Fig. 1b). The diameter of the colony was 17 mm after 7-day incubation at 30 °C. Microscopic morphology showed that the hyphae were closely septated with thick and darkened septa; the conidia were globose to subglobose and brown to dark brown in colour, each with a prominent scar at the end of the conidium and ramoconidia (Fig. 1c,d). The identity of UM 843 described previously^8^ is further confirmed with additional scanning electron microscopy (SEM) and internal transcribed spacer (ITS)-based phylogenetic tree analysis. Under the SEM, coronate conidia showed characteristics described by Dugan *et al.*^9^, i.e. they were protuberant, thickened, darkened with a central convex dome and surrounded by a raised periclinal rim. The verruculose ornamentation of conidia and ramoconidia in UM 843 corresponded to the typical morphology of *C. sphaerospermum* (Fig. 1e–g)^1^. The ITS-based phylogenetic tree showed UM 843 to be tightly grouped with the *C. sphaerospermum* cluster (Fig. 2).

### Genomic sequencing and assembly

A total of 19,253,334 sequencing reads of a 500-bp insert library (1.7 Gb) generated from previous study^8^ and 9,987,556 reads of a 5-kb insert library (899 Mb) generated in the present study were used in *de novo* assembly (Table 1). The combined 2,632 Mb sequenced reads represents ~98-fold depth of genome sequence coverage.

### Transposable elements

We identified 284,298 bases (1.06% of the genome size) as putative transposable elements, with 231 class I retrotransposons and 82 class II DNA transposons (Table 2). As in other reports^10^, Gypsy element is the most frequent (31.95%). Among the class II DNA transposons is a rare Crypton transposable element reported so far, in only eleven pathogenic fungi^11^. UM 843 is the only Dothideomycetes so far predicted to contain the Crypton transposon that may be involved in DNA integration.

### Gene annotation

**Figure 3:**
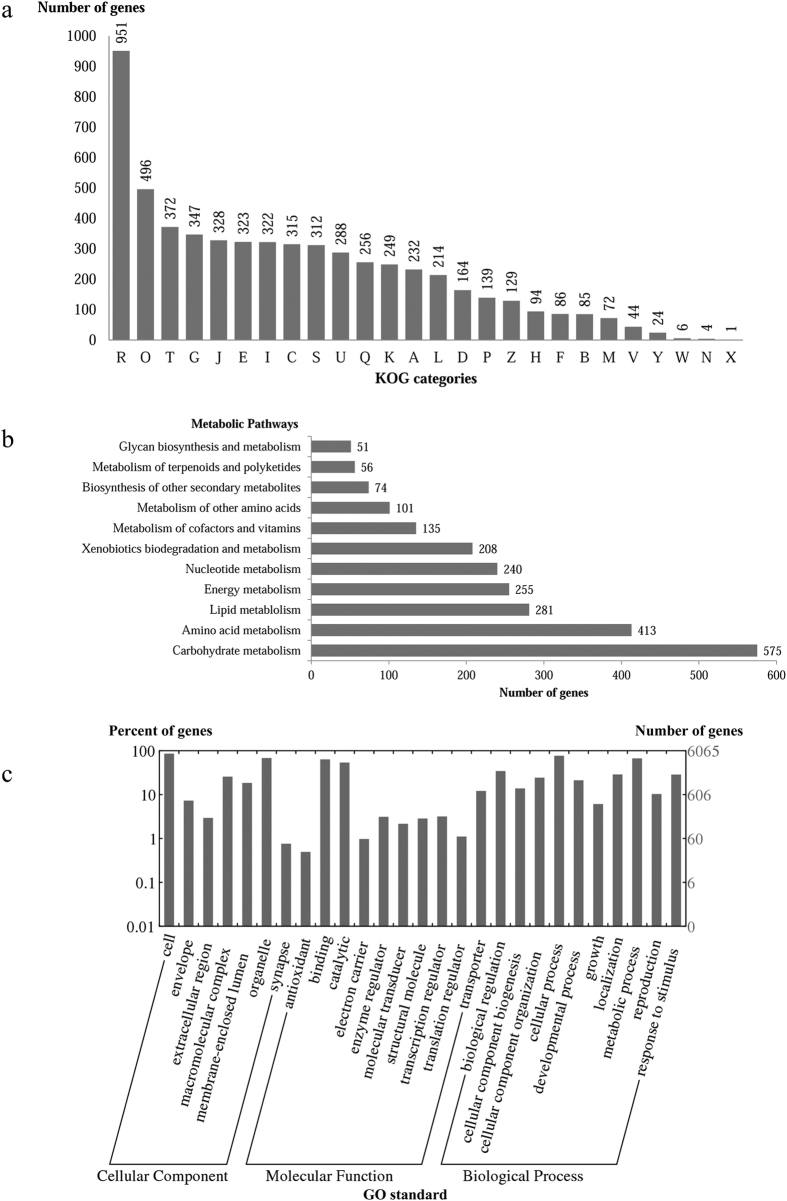
KOG, KEGG and GO classifications of predicted genes in *C. sphaerospermum* UM 843. Distribution of KOG classes (**a**), KEGG metabolic pathway (**b**), and GO annotations in UM 843. A, RNA processing and modification; B, Chromatin structure and dynamics; C, Energy production and conversion; D, Cell cycle control, cell division, chromosome partitioning; E, Amino acid transport and metabolism; F, Nucleotide transport and metabolism; G, Carbohydrate transport and metabolism; H, Coenzyme transport and metabolism; I, Lipid transport and metabolism; J, Translation, ribosomal structure and biogenesis; K, Transcription; L, Replication, recombination and repair; M, Cell wall/membrane/envelope biogenesis; N, Cell motility; O, Posttranslational modification, protein turnover, chaperones; P, Inorganic ion transport and metabolism; Q, Secondary metabolites biosynthesis, transport and catabolism; R, General function prediction only; S, Function unknown; T, Signal transduction mechanisms; U, Intracellular trafficking, secretion, and vesicular transport; V, Defence mechanisms; W, Extracellular structures; X, Unnamed protein and Z, Cytoskeleton.

The 9,652 predicted genes in UM 843 were mapped to the KOG, KEGG and GO (Table 1; Fig. 3). A total of 5,215 predicted proteins were annotated redundantly into 5,853 KOG classifications (Fig. 3a). Among the highest annotated groups, posttranslational modifications of proteins appeared significant in cellular regulation, development and adaptation to stress^12^. We identified 69 putative genes encoding chaperones in group O (Posttranslational modification, protein turnover, chaperons) which may be associated with stress adaptation, misfolded proteins degradation via the ubiquitin-proteasome system, regulatory degradation of metabolic enzymes and cell viability. In group G (Carbohydrate transport and metabolism), the largest number of genes (77) is for a putative permease of the major facilitator superfamily. Furthermore, 26 were putative monocarboxylate transporters which are important to energy utilisation, intracellular pH regulation, and virulence in some pathogenic fungi^13^.

The top five metabolic pathways of the genes annotated in KEGG pathways were carbohydrate metabolism (575), amino acid metabolism (413), lipid metabolism (281), energy metabolism (255), and nucleotide metabolism (240) (Fig. 3b). It is not surprising for UM 843 to contain many genes involved in carbohydrate metabolism since carbon source is an essential nutrient for fungal growth, conidiation and virulence^14^.

Based on the GO classifications, 6,065 predicted genes received a GO assignment (Fig. 3c). A total of 1,733 and 1,080 genes were assigned to the response to stimulus category (GO: 0050896) and the response to stress category (GO: 0006950), respectively. The genes were further annotated to various stress responses. The highest number of predicted genes were assigned to the response to osmotic stress category (139). This distribution of genes might be reflective of the habitats of *C. sphaerospermum* in which the fungus has to combat with osmotic imbalance^1^. The osmotic responses of *C. sphaerospermum* UM 843 are further discussed in the subsection of “Fungal adaptation and stress responses”.

### Gene families

**Figure 4:**
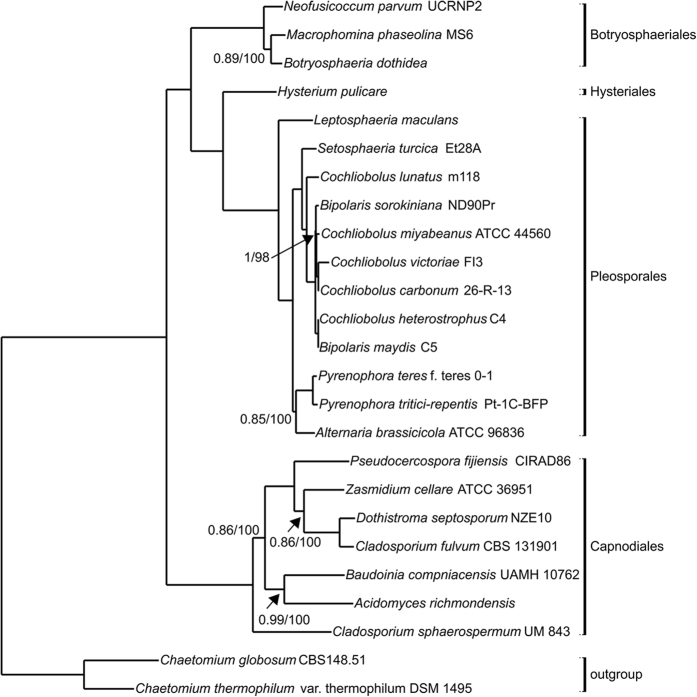
Phylogenomic tree of *C. sphaerospermum* UM 843 and 22 fungi under class Dothideomycetes. The phylogenomic tree was constructed with 23 Dothideomycetes spp. including UM 843 and two outgroups from Sodariomycetes spp. using Bayesian and maximum likelihood analysis. The first number at the node is Bayesian posterior probability followed by the maximum likelihood bootstrap number. Values less than 1 or 100 for posterior probability and maximum likelihood bootstrap number, respectively, were shown on branches.

We performed all-against-all BLASTP for 303,264 proteins from 23 Dothideomycetes and two Sordariomycetes as the outgroup (Supplementary Table S1), obtaining 24,581 orthologous clusters with 2,203 single-copy orthologues (one copy of gene from each species). Maximum likelihood and Bayesian trees were constructed using concatenated alignments generated from 10% of the single-copy orthologues identified (220 single-copy orthologues). The topology of the maximum likelihood tree built by RaxML was identical to that of the Bayesian tree. The Dothideomycetes were categorised into four orders encompassing Pleosporales, Capnodiales, Botryosphaeriales and Hysteriales. The UM 843 genome is within the order Capnodiales where it forms a sister-group relationship with two clusters (Fig. 4).

Of the 23,800 orthologous clusters generated from the 23 dematiaceous Dothideomycetes, 3,333 clusters (14%) were conserved within the class while 51 clusters contained 125 UM 843 unique genes (recent paralogues) in this study set (Supplementary Table S2). Hydrophobins (DOTH 13561), mitogen-activated protein kinases (MAPKs) (DOTH 14960) and other metabolism related genes were among the UM 843-specific putative genes found. The three putative genes from DOTH 14960 shared ≥60% similarity with *Schizosaccharomyces pombe* MAPK *Spk1* (*Fus3* orthologue) (GenBank: P27638). This protein is responsible for appressorium formation and pathogenicity of certain plant pathogenic fungi, sexual/asexual reproduction, hyphal growth and conidial germination in filamentous fungi^15^. However, these genes might be atypical MAPKs that were identified in *Fusarium graminearum*^16^ as the typical T-X-Y motif in the activation loop of MAPK was absent. However, the exact role of these atypical MAPKs remain unknown.

### Sexual reproduction

To date, *Cladosporium herbarum* and *Cladosporium silenes* are species with established anamorph-teleomorph stages while *Cladosporium grevileae* is only known with a sexual stage^17^. Most of the genes involved in sexual reproduction were identified in UM 843 (Supplementary Table S3). This strain might be a heterothallic fungus with a predicted high-mobility-group (HMG) domain containing *Mat1-2* gene (UM843_3044), sharing 65.05% identity with *Dothistroma septosporum Mat1-2* (GenBank: ABK91354). This suggested that sexual reproduction might occur in *C. sphaerospermum*. The gene was found in adjacent to genes encoding DNA lyase (Apn2), anaphase promoting complex protein and cytochrome c oxidase subunit Vla (Cox13). The presence of *Apn2* and *Cox13* nearby *Mat1-2* is similar to the previously reported mating type cluster^18^ (Supplementary Fig. S1). Furthermore, although the genes involved in mating and cell cycle in fungi are initiated by Fus3 MAPK signalling pathway which is stimulated by pheromones^19^, no pheromone genes were predicted in UM 843. These features posed a possibility that this fungal strain is unable to carry out mating process despite the presence of sexual reproduction genes. Nonetheless, it has been shown that some fungi can undergo sexual reproduction without the activation of pheromone response pathway by pheromone ligand^19^. Hence, it is still possible that UM 843 can mate by a different mechanism of pheromone activation or without pheromone activation.

### Carbohydrate Active enZymes (CAZymes)

Fungal CAZymes play an important role in the degradation of the plant cell wall into carbon sources required for fungal growth or the infection of the plant host^20^. In this study, a total of 605 putative CAZyme catalytic domains comprising 261 glycoside hydrolases (GH), 98 glycosyltransferases (GT), 114 carbohydrate esterases (CE), 14 polysaccharide lyases (PL), 77 auxiliary activities (AA), and 41 carbohydrate-binding modules (CBM) were identified in UM 843 (Supplementary Table S4). The CAZymes predicted in all Dothideomycetes genomes were compared with those in UM 843 for correlation with its possible lifestyle (Supplementary Fig. S2). Zhao *et al.*^20^ revealed that CE11, GH73, GH80 and GH82 families were absent in saprophytic fungi. This absence was also observed in UM 843 suggesting it to be a saprophyte. However, at this stage, no conclusive inference can be drawn that UM 843 belongs to the saprophytic group.

UM 843 contains at least 171 CAZymes that are involved in plant cell wall degradation (Supplementary Table S5). The presence of a high number of hemicellulose and pectin degrading CAZymes suggested the preference of this fungus for soft plant tissue^21^ (Supplementary Table S5). Nevertheless, UM 843 has the highest number of predicted CBM1 (carbohydrate-binding modules 1) among the Capnodiales (Supplementary Table S6). Apart from associating cellulases in ensuring contact between catalytic domain and substrate, CBM1 has been shown to be able to disrupt the crystalline structure of cellulose by non-hydrolytic cleavage of inter- and intra- hydrogen bonds of polysaccharide chains^22^. The weakened cellulose structure allows easy accessibility of other enzymes such as hemicellulolytic enzymes to carry out catalytic reactions.

### Peptidases

In UM 843, 130 predicted peptidases were identified with no predominance of any particular enzyme family (Supplementary Fig. S3; Supplementary Table S7). This observation is consistent with the saprophytic lifestyle to degrade different types of substrate complexes available in the environment into smaller residues to be absorbed into the fungal cell^23^.

The small sized (2–5 × 2–4 μm) *C. sphaerospermum* conidia is easily disseminated, and hence, may be inhaled by humans to reach the lung alveoli^1^. We postulated reactions involving secreted peptidases to take place when the conidia of *C. sphaerospermum* reach the lungs of humans. Five of the 31 secreted (two A01, two S09, one M36) and one non-secreted peptidase from the A01 family in UM 843 were shown to be putative peptidases involved in lung tissues disruption. These are the A01 secreted aspartic peptidases (UM843_1326 and UM843_4966) belonging to the holotype peptidase F (51.70% and 61.99% identity, respectively) known to hydrolyse elastin and laminin, which are the components of lung^24^; a secreted metallopeptidase from family M36 (UM843_2925) showing 67.86% identity to a holotype fungalysin involved in elastin hydrolysis^25^; and a cell wall-associated aspartic peptidase, PEP2. This PEP2 peptidase (UM843_5823; 76.15% identity) facilitates the penetration of young hyphae into the host connective tissue^26^.

Two peptidases (UM843_2883 and UM843_1649) belonging to the family S09 showed 56.24% and 50.07% identity to dipeptidyl peptidase (DPP) IV and DPP V, respectively. These two peptidases have the Gly-X-Ser-X-Gly conserved sequence motif and catalytic triad Ser 631, Asp 711, His 746 and Ser 565, Asp 646, His 678 in UM843_2883 and UM843_1649 respectively. The predicted catalytic sites are conserved with other reported DPPs (Supplementary Figs S4 and S5). DPP IV has been shown to facilitate colonisation of the lung by binding and subsequently degrading the dipeptide of collagen^27^, whereas DPP V is the elicitor of host defence mechanisms^28^. These peptidases might work in concert to disrupt lung tissues.

### Secondary metabolites

We predicted 16 secondary metabolite backbone genes in UM 843 (Supplementary Table S8). One polyketide synthase-nonribosomal peptide synthase (PKS-NRPS) hybrid, five PKS or PKS-like and ten NRPS or NRPS-like enzymes were annotated using SMURF analysis. Among the PKS, two reducing PKS were predicted to contain the domain arrangements of ketosynthase (KS)-acyltransferase (AT)-dehydratase (DH)-methyltransferase (ME)-enoyl reductase (ER)-ketoreductase (KR)-acyl carrier protein (ACP) (UM843_7344) and KS-AT-DH-ER-KR-ACP (UM843_9325). One of the non-reducing PKS (UM843_1729) was found likely to be involved in pigment synthesis and two NRPS (UM843_7306 and UM843_8410) are likely to be responsible for siderophore biosynthesis.

#### Melanin

Most fungi synthesise melanin via the 1,8-dihydroxynaphthalene (DHN) biosynthesis pathway^29^ to protect them from UV irradiation, desiccation, high temperatures, and oxidants. Evidence for this pathway in *C. sphaerospermum* was shown by the generation of melanin-deficient *C. sphaerospermum* from cultures in medium containing tricyclazole, an inhibitor of DHN melanin biosynthesis^30^. The PKS gene (UM843_1729) we predicted in UM 843 is best matched to a characterised conidial yellow pigment biosynthesis PKS from *Aspergillus fumigatus* (*alb-1*) (GenBank: Q03149)^31^ and shows high identity to a predicted *Cladosporium phlei* Cppks1 protein (GenBank: AFP89389) involved in pigment biosynthesis^32^ (Supplementary Table S9). A starter unit: ACP transcylase (SAT) domain (PF16073) typical of non-reducing PKS was identified in the gene^33^. Also predicted were domains in the order of SAT-KS-AT-DH-ACP-ACP-TE which was similar to that of Alb-1 and Cppks1. We also predicted a scytalone dehydratase (UM843_148) and two tetrahydroxynaphthalene reductase genes (UM843_1726; UM843_7560) that are involved in DHN melanin biosynthesis. Interestingly, one of the THN-reductases (UM843_1726) was found in a cluster with the PKS gene (UM843_1729) and a gene encoding transcription factor Cmr1 (UM843_1727) (Supplementary Fig. S6). This cluster was previously reported in *Cochliobolus heterostrophus* and *Alternaria brassicicola*^34^ but in a different gene orientation and organisation. Recently, some fungi were shown to synthesise the key melanin precursor pentaketide 1,3,6,8-tetrahydroxynaphthalene (T4HN) with an additional polyketide precursor post-modification step by the yellowish green-1 (*yg-1)* gene^31^,^35^. In this work, we identified two *yg-1* like genes annotated as *Wdyg-1* (UM843_912) and *Ayg-1* (UM843_6732) in the UM 843 genome (Supplementary Table S9). In *Exophiala dermatitidis*, *Wdyg-1* deacetylates 2-acetyl-1,3,6,8-tetrahydroxynaphthalene to T4HN^35^ while the *A. fumigatus Ayg-1* modifies the product of *alb-1* by removing acetoacetic acid to produce T4HN^36^; this process might also occur in UM 843. Thus, we hypothesise that UM 843 might synthesise DHN melanin with an additional post-modification step.

#### Siderophores

Iron plays important roles in cellular processes but excessive of iron in cells is dangerous to the organism. To overcome the bioavailable scarcity and cytotoxic effect of iron, fungi have developed different strategies for iron uptake and regulation. Siderophore-mediated Fe^3+^ uptake is one of the mechanisms for iron homeostasis^37^. We found putative genes that are essential in the synthesis of siderophores (Supplementary Table S10). UM843_8412 and UM843_7304 are putative genes encoding L-ornithine-N^5^-monooxygenese involved in the first committed step in siderophore biosynthesis while UM 843_7306 encodes NRPS SidD that is responsible for fusarinine-type siderophore biosynthesis^38^. As previously reported by Schrettl *et al.*^38^, our analysis showed that UM843_7306 has a domain arrangement of adenylation (A)-thiolation (T)-condensation (C)-T-C, similar to that in *A. fumigatus* SidD (GenBank: Q4WF53). The clustering of siderophore biosynthesis genes in UM 843 appears to be different from the gene clusters reported by others^39^, probably as a result of rearrangement during genome evolution and speciation in fungi. In UM 843, the putative gene cluster encompasses genes encoding L-ornithine-N^5^-monooxygenase, esterase, NRPS SidD, ABC transporter SitT, carnitinyl-CoA dehydratase and acetyltransferase SidF (Supplementary Fig. S7). However, the orthologue of *SidG* gene that is responsible for the synthesis of triacetylfusarinine C (TAFC) was absent. Thus, UM 843 may be synthesising only the TAFC precursor, fusarinine C, but not TAFC itself. Nevertheless, UM 843 possess the ability to utilise TAFC produced by other organisms. A gene encoding siderophore esterase (UM843_1515) similar to *EstB* of *A. fumigatus* (GenBank: XP748686) that hydrolyses iron-chelated TAFC was identified. This gene was located adjacent to the TAFC transporter *MirB* (UM843_1516) (Supplementary Fig. S8), showing an organisation similar to that reported previously^39^.

Another putative gene UM843_8410 was found to be involved in the production of ferrichrome-type siderophores. Ferrichrome NRPSs have a diversity of domain architectures consisting of two to four complete A-T-C modules which are usually followed by a T-C repeat^37^. The gene UM843_8410 has the order of domains A-T-C-A-T-C-T-C-A-T-C-T-C-T-C. The gene organisation of the ferrichrome-type siderophore in UM 843 is similar to that in *C. heterostrophus*^37^, where the gene encoding an ABC transporter spans between L-ornithine-N^5^-monooxygenase and ferrichrome NRPS encoding genes (Supplementary Fig. S9).

#### PKS-NRPS Hybrid

Recently, an isolate of *C. sphaerospermum* was reported to synthesise polyketide hybrid cladosins such as Cladosin C showing mild antiviral activity^40^. In UM 843, a putative PKS-NRPS hybrid, UM843_7284, shared 40% and 41% identity with *Talaromyces stipitatus* putative PKS (GenBank: XP_002478535) and *Aspergillus clavatus* PKS-NRPS cytochalasin (GenBank: A1CY8), respectively. The domain arrangement of the gene revealed an arrangement of KS-AT-KR-ACP-C-A-T-reductive domain (R), which is different from that of *T. stipitatus* and *A. clavatus*. Further inspection of the predicted gene cluster revealed neighbouring genes that might be associated with the synthesis and transport of the product, such as genes encoding transporters, cytochrome P450, alpha beta hydrolase, thioesterase and transcription factor domain containing proteins (Supplementary Table S11). As only one putative PKS-NRPS hybrid was identified in the genome, further studies need to be carried out to confirm the function of this gene in the synthesis of cladosins.

### Fungal adaptation and stress responses

Most fungi have their own system to respond to multiple stresses from their ecological niche for adaptation and survival. In order to survive in hostile environments, they have to be able to detect stress, transduce stress signals and respond to the stress^41^. We found 340 genes involved in stress responses including amino acid starvation, nitrogen starvation, iron starvation, osmotic stress, oxidative stress, and heat stress (Supplementary Table S12).

Apart from biosynthesis of sidorephores in iron acquisition, UM 843 might also be employing another high-affinity iron acquisition system, the reductive iron assimilation mechanism (RIA), to regulate iron homeostasis. We identified a ferroxidase and iron permease similar to FetC (UM843_5150, 69.12% identity to P38993) and Ftr1 (UM843_5151, 59.69% identity to P40088), respectively, that are involved in the RIA. They were located adjacent to each other in the UM 843 genome (Supplementary Fig. S10). The ferroxidase and iron permease encoding gene cluster was also found in other fungi^37^.

#### Adaptation in hypersaline environment

*C. sphaerospermum* has also been isolated consistently from hypersaline environment^1^. In this study, UM 843 is shown able to grow at a high concentration of NaCl (20% w/v) (Supplementary Fig. S11). UM 843 was predicted to contain 21 genes encoding plasma membrane and intracellular cation transporters which are involved in cation homeostasis by maintaining low intracellular Na^+^ concentration. Also identified were the plasma membrane H^+^- ATPases that supply energy to the secondary transporters (Table 3; Supplementary Table S13) and all the subunits of V-type ATPase complex that play an important role in acidification of vacuolar lumen and correct functioning of other organelles (Supplementary Table S14). The V-type ATPase complex also works together with the plasma membrane H^+^- ATPase to maintain the cytosolic pH homeostasis^42^. As seen in Table 3, UM 843 contains more *Ena* genes than *Nha1* genes. This might confer the ability to survive in the near neutral to alkaline pH of a hypersaline environment, as Ena is important in the export of Na^+^ ions at alkaline pH^43^. However, *Nha1* is also required by the fungus as this gene is critical in the immediate response to osmotic shock^44^.

A comparison of the UM 843 transporters with those reported in halotolerant *Hortaea werneckii*, halophilic *Wallemia ichthyophaga* and non-halotolerant *Mycosphaerella graminicola* was carried out to determine the genes confer to the ability of UM 843 to survive in high salinity environment (Table 3). UM 843 contains more genes encoding Trk, Tok, Ena and Pho89 compared to *M. graminicola* although it has lesser genes compared to *H. werneckii* which had undergone recent whole genome duplication^45^ (Table 3). In addition, we managed to identify genes encoding K^+^(Na^+^)-ATPase (alkali cation uptake, Acu transporters) and K^+^-H^+^ symporter (Hak symporters) in UM 843 which were not found in *H. werneckii*. These transporters function in high affinity uptake of K^+^ ions which might be beneficial for organisms to adapt in a hypersaline environment^46^. It has been reported that different strategies were used by *H. weneckii* and *W. ichthyophage* to counteract high salinity^45^,^47^. The diversity of cation transporters found in UM 843 indicates that this fungus possibly uses strategies that are different from those used by *H. werneckii* and *W. ichthyophaga* in their response to dynamic changes in salinity.

Besides the accumulation of nontoxic ions to overcome osmotic stress, the accumulation of compatible solutes is another strategy employed by microorganisms in osmo-adaptation. Glycerol was reported as the main solute used by *H. werneckii* and *W. ichthyophaga* to maintain cell turgor pressure^48^. The synthesis of glycerol is carried out by NAD-dependent glycerol-3-phosphate dehydrogenase (Gpd) and glycerol-3-phosphatase (Gpp), acting on the glycolysis intermediate dihydroxyacetone phosphate. The putative glycerol metabolism-related genes in UM 843 are listed in Supplementary Table S12. The *Gpd* gene (UM843_9164) shared 74.22% and 73.29% identity with *H. werneckii Gpd1A* and *Gpd1B*, respectively. Similar to the *Gpd* of *H. werneckii* and *W. ichthyophaga*, UM843_9164 does not have the N-terminal type 2 peroxisomal targeting sequence (PTS2) (Supplementary Fig. S12). The absence of PTS2 in the gene is suggested to be an advantage for survival in high salinity environments^49^.

In addition, six putative genes were identified to encode glycerol/H^+^ symporter Stl1 that is essential for active glycerol uptake. All the genes appeared to contain 12 transmembrane domains, a typical characteristic of Stl1^50^ (Supplementary Fig. 13). The glycerol/H^+^ symporter located in the plasma membrane actively imports glycerol into the cell during hyperosmotic shock^50^. Moreover, three genes encoding aquaglyceroporin (UM843_5638, UM843_4817 and UM843_4886) that function in the efflux of glycerol during hypoosmotic conditions were also identified. The presence of sugar/H^+^ symporters and aquaglyceroporins in large numbers suggests that UM 843 can mount a rapid response to combat a highly dynamic concentration of external NaCl. Additionally, UM 843 might synthesise taurine to act as an osmoregulant (Supplementary Fig. S14), as suggested in the acidophile *Acidomyces richmondensis*^51^.

The high osmolarity glycerol (HOG) pathway is an important mitogen activated protein kinase (MAPK) signalling pathway that is involved in osmoregulation activated under osmotic and cationic stress^15^. We detected the genes involved in the HOG signalling pathway in UM 843 (Supplementary Table S12). This pathway is activated by two types of osmosensors, the Sho1 (UM843_8679) and Sln1 (UM843_4487). The histidine kinase Sln1 together with the phosphorelay molecule Ypd1 (UM843_8084) and response regulator Ssk1 (UM843_8730) form the final receptor that activates the MAPK kinase Pbs2 (UM843_2812) and in turn, phosphorylates MAPK Hog1 (UM843_5411). In the activation via the Sho1 branch, Sho1 activates Pbs2 through Ste11 and Ste20 (UM843_3491)^15^. The *Hog1* gene of UM 843 (UM843_5411) shared 92.48% identity with *H. werneckii Hog1*. The sequence alignment of *Hog1* from UM 843, *H. werneckii*, *A. fumigatus* and *S. cerevisiae* showed a conserved T-G-Y phosphorylation motif in the activation loop and a common docking domain-containing conserved YHDP[T/S]DEP motif (Supplementary Fig. S15). The negatively charged amino acids in the YHDP[T/S]DEP motif (underlined) are important for the interaction of MAPK with downstream effectors^52^. The activation of HOG signalling pathway leads to osmotic adaptation via several responses such as the synthesis of glycerol via activation of *Gpd1* and regulating the expression of *Stl1*, *Nha1*, *Tok1* and *Ena1*^44^,^53^. Four HOG regulated genes encoding PMP3 that is responsible for cationic stress response via cell membrane potential modulation were also identified in UM 843^54^. The activation of the HOG signalling pathway, regulation of transporters and synthesis of various compatible solutes in UM 843 mediate salt tolerance in the fungus.

### Fungal allergens

Allergy is one of the main concerns in medical mycology as numerous fungi such as *Aspergillus*, *Alternaria*, *Penicillium*, and *Cladosporium* are known to cause allergic reactions^6^. Among *Cladosporium* species, *C. herbarum* is the best studied with a total of 60 antigens, of which at least 36 having reactions with IgE antibodies from patients’ sera have been identified, as reported in the latest Thermo Scientific allergen database (http://www.phadia.com/en/Products/Allergy-testing-products/ImmunoCAP-Allergen-Information/Molds-and-other-Microorganisms/Allergens/Cladosporium-herbarum-/). Although *C. sphaerospermum* has been reported to be an allergy-causing mould, specific allergens have not been identified^55^. BLASTX similarity searches using the predicted gene models identified 28 genes functionally annotated as allergens showing >50% identity to the respective allergen (Table 4). Although not all the predicted genes will encode allergenic proteins, this feature indicates a high potential for serum cross-reactivity in patients sensitised with these fungal allergens, as proteins with >50% identity to known allergens are likely to cross-react^56^. For instance, cross reactivity among fungal allergens has been reported between Cla h 8 and Alt a 8^57^; and Cand b 2 and Asp f 3^58^.

Compared to other Dothideomycetes, four predicted gene families encoding allergens, *viz.* Cla h 10, Cla h HCh 1, Asp f 23 and Cand a 1, have higher copy numbers of genes in UM 843 (Supplementary Table S15). Interestingly, Cla h HCh 1 was only found in UM 843 (UM843_1201, UM843_6061, UM843_4115 and UM843_3639). These putative proteins were similar to *C. herbarum* Cla h HCh 1 (GenBank: Q8NIN9) (Table 3). The mRNAs of the four genes (UM843_1201, UM843_6061, UM843_4115 and UM843_3639) were detected by RT-PCR (Supplementary Fig. S16). The extracted mRNA used for cDNA synthesis were of high quality (Supplementary Fig. S17) and the RT-PCR products corresponding to the size of open reading frames (ORF) (~300 bp) were successfully amplified from the converted cDNA. This finding revealed that these predicted allergen-encoding genes are not pseudogenes but were expressed in UM 843 (Supplementary Figs S16, S18–S21). Weichel *et al.*^59^ showed that Cla h HCh1 was the only hydrophobin in *C. herbarum* that elicited a specific IgE-dependent allergic reaction. As previously reported^60^, we noticed that UM843_1201, UM843_6061, UM843_4115 and UM843_3639 contain eight conserved cysteine residues each (Supplementary Fig. S22). It would be interesting to determine the allergenicity of Cla h HCh1 in *C. sphaerospermum* as it might be a *Cladosporium-*specific allergen.

The *C. sphaerospermum* UM 843 genome provides the first glimpse into the genetic basis of important adaptation traits for the fungus to survive in diverse environmental niches. The same genes that enable adaptation to adverse environmental conditions may also confer a selective advantage for survival and adaptation in adverse microenvironments in human hosts. The isolation of UM 843 from the peripheral blood sample of a patient foretells the emergence of *C. sphaerospermum* as an important opportunistic pathogen in susceptible human populations. Furthermore, allergen-encoding genes identified in this study could be further validated at the protein level and tested for specific allergenicity. These candidate allergens could be useful for the development of immunotherapeutic vaccines against allergic fungal reactions in humans.

## Methods

### Ethics statement

The genome used in this study was obtained from a fungal isolate routinely cultured and archived by the mycology laboratory in a teaching hospital^7^. The authors were not involved in the specimen collection and related clinical information was not accessible. In such circumstances, ethical clearance is exempted from the University of Malaya Medical Centre (UMMC) Medical Ethics Committee for this study.

(http://umresearch.um.edu.my/doc/File/UMREC/6_CODE%20OF%20RESEARCH%20ETHICS%20%20IN%20UNIVERSITY%20OF%20MALAYA.pdf).

### Fungal isolate

UM 843 was isolated from the peripheral blood sample of a patient in UMMC, Malaysia. The isolate was sub-cultured on Sabouraud Dextrose Agar (SDA). For the scanning electron microscopy (SEM), a 7-days old culture on SDA was processed and viewed under SEM (Phillips XL30 ESEM, the Netherlands). To test the ability of UM 843 to grow in high salt medium, the isolate was cultured in Sabouraud Dextrose Broth (SDB) supplemented with 5%, 10%, 15%, 20% and 25% (w/v) of NaCl. The growth of the fungus was observed up to 14 days of incubation.

### Molecular identification

ITS-based molecular identification was carried out using ITS region. The isolate was subjected to DNA extraction, amplification, DNA sequencing and ITS-based phylogenetic analysis was performed as previously described with slight modification^7^. The selection of species to be included in the phylogenetic analysis was limited to species closely related to *C. sphaerospermum*. Complete ITS1-5.8S-ITS2 sequences for 18 *C. sphaerospermum* species complexes and two outgroup strains were obtained from GenBank for phylogenetic tree construction. Bayesian tree analyses were performed using MrBayes. Bayesian Markov Chain Monte Carlo (MCMC) analysis was conducted by sampling across the entire general time reversible (GTR) model space. A total of 150,000 generations were run with a sampling frequency of 100, and diagnostics were calculated for every 1,000 generations. A burn-in setting of 25% was used to discard the first 375 trees.

### Genome analysis workflow of *Cladosporium sphaerospermum* UM 843

The genomic DNA extraction, sequencing, assembly, gene model prediction, and functional annotation of *C. sphaerospermum* UM 843 was conducted as previously described^61^ with some modifications. The 5-kb insert library was sequenced using the Illumina HiSeq 2000 system. The sequenced reads were then combined with the 500-bp Illumina sequenced reads^8^ for further processing. Genes associated with stress responses were identified by performing a local BLASTP search against a database built from the Fungal Stress Response Database (FSRD, http://internal.med.unideb.hu/fsrd) using the criteria of e-value threshold ≤1e-5, identity exceeding 50% and subject coverage exceeding 70%.

The protein sequences of all current publicly-available dematiaceous Dothideomycetes genomes were downloaded from different databases to determine the orthologues in UM 843 for the orthologous genes and genome comparative analysis (Supplementary Table S1). A phylogenomic tree was constructed using all proteome clusters of Dothideomycetes generated from comparative analysis with inclusion of another two proteomes from class Sordariomycetes as outgroup strains (Supplementary Table S1). A total of 220 single-copy orthologous genes containing one member in each species was subjected to individual sequence alignments and removal of spurious sequences or poorly aligned regions with trimAL (with the *automated* option). The filtered multiple alignments were then concatenated into a superalignment with 110,781 characters. Subsequently, the best-fit substitution model was selected by running ProtTest version 3.2^62^ with AIC calculation on the alignment. The phylogenomic tree was constructed by using MrBayes as previously described^61^ and RAxML version 7.7.9^63^. The MCMC was run with a sampling frequency of 100 for 250,000 generations, and burn-in setting of 25%. RAxML was run with model PROTGAMMALGF to search for the best-scoring maximum likelihood tree, followed by 100 bootstrap replicates. Convergence was observed after 50 replicates using -I autoMRE option in RAxML.

### RNA extraction and Reverse Transcription PCR of hydrophobin genes

The 7-days old mycelia were scraped off from the agar surface and 100 mg of samples were crushed into fine powder with liquid nitrogen. Total RNA were then isolated from the frozen samples using RNeasy Plant Mini Kit (Qiagen, Germany) according to the manufacturer’s protocol. The concentration of RNA was determined using spectrophotometer at the wavelength of 260 nm. The purity of RNA sample was assessed using the ratio of absorbance at 260 nm and 280 nm. The size distribution and integrity of the RNA were checked by 1% (w/v) agarose gel electrophoresis. The RNA bands were visualised by RedSafe nucleic acid staining solution (Intron Biotechnology, Korea) staining and observed under UV light. The RNA samples with the ratio of absorbance at 260 nm and 280 nm was between 1.8 and 2.1 and high integrity were used for subsequent cDNA synthesis. cDNA was synthesised from the extracted total RNA using RevertAid H Minus first strand cDNA synthesis kit (Fermentas, Germany) according to manufacturer’s protocol with only modification in using a primer mixture of 0.5 μL oligo (dT)_18_ primer and 0.5 μL random hexamer in the cDNA synthesis. The primers used for amplification were listed in the Supplementary Table S16. PCR was performed with an initial denaturation at 95 °C for 5 min, followed by 35 cycles of denaturation at 95 °C for 30 seconds, annealing at 58 °C for 30 seconds, extension at 72 °C for 1 min, and final extension at 72 °C for 10 min. The PCR product was then electrophoresed in 1% (w/v) agarose gel at 90V for 30 min, purified and sent for Sanger sequencing (First Base Laboratories, Malaysia).

## Additional Information

**How to cite this article**: Yew, S. M. *et al.* Insight into different environmental niches adaptation and allergenicity from the *Cladosporium sphaerospermum* genome, a common human allergy-eliciting Dothideomycetes. *Sci. Rep.* **6**, 27008; doi: 10.1038/srep27008 (2016).

## Supplementary Material

Supplementary Dataset 1

Supplementary Dataset 2

## Figures and Tables

**Table 1:** Genome features of *C. sphaerospermum* UM 843 generated from combined insert libraries.

	*C. sphaerospermum* UM 843
Reads from 500 bp insert library (Mb)	1,733
Reads from 5 kb insert library (Mb)	899
Total Reads (Mb)	2,632
Assembly size (bp)	26,644,473
Number of contigs (≥200 bp)	867
Contigs size (N50) (kb)	92,815
Number of scaffolds (≥1000 bp)	155
Scaffolds size (N50) (bp)	969,659
G+C content (%)	55.32
Number of predicted genes (≥99 bp)	9,652
Average gene length (bp)	1,482
Average number of exons per gene	2.26
rRNA	42
tRNA	196
KEGG	999
GO	6,065
KOG	5,853
Pfam	6,655

**Table 2:** Transposable elements predicted in *C. sphaerospermum* UM 843 genome.

Class	Family Name	Total Number	Total Bases	Percentage of genome assembled
I	DDE_1	42	43,548	0.16%
	gypsy	100	63,804	0.24%
	LINE	61	90,510	0.34%
	ltr_Roo	2	1,191	0.00%
	TY1_Copia	26	24,141	0.09%
II	cacta	4	609	0.00%
	Crypton	1	258	0.00%
	hAT	37	40,137	0.15%
	helitronORF	3	2,061	0.01%
	mariner	6	2,355	0.01%
	mariner_ant1	10	4,890	0.02%
	MuDR_A_B	21	10,794	0.04%
Total		313	284,298	1.06%

**Table 3:** Putative transporters involved in high salinity survival in *C. sphaerospermum* UM 843, *W. ichthyophaga*, *H. werneckii*, and *M. graminicola*.

Cellular location	Transporter type	substrate specificity	^a^*Sc*homologue	UM 843	^b^*Wi*^1^	^c^*Hw*^2^	^d^*Mg*^2^
Plasma membrane	Uniporter	K^+^ uptake	Trk1, 2	2	1	8	1
	Channel	K^+^ efflux	Tok1	2	0	4	1
	P-type ATPase	K^+^ influx	Acu	1	2	0	1^3^
	Symporter	K^+^ influx	Hak	1	0	0	2^3^
	Antiporter	Na^+^/H^+^ exhange	–	3	1	–	–
	Antiporter	Na^+^, (K^+^)/H^+^ exhange	Nha1	2	2	8	3
	P-type ATPase	Na^+^ (and Li^+^) efflux	Ena1,2,5	4	2	4	3
	P-type ATPase	H^+^ export	Pma1	1	3	4	–
	Symporter	Na^+^/Pi cotransporter	Pho89	3	1	6	2
Vacuole	V-type ATPase	H^+^ uptake	Vma1	1	1	2	–
	Antiporter	Na^+^, K^+^/H^+^ exchange	Vnx1	0	1	2	1
Endosomal	Antiporter	Na^+^/H^+^ exchange	Nhx1	1	1	2	1
Golgi apparatus	Antiporter	K^+^/H^+^ exchange	Kha1	1	2	2	1
Mitochondria	Antiporter	K^+^/H^+^ exchange	Mrs7/Mdm38	1	1	2	1

^a^*S. cerevisiae*

^b^*W. ichthyophaga*.

^c^*H. werneckii*.

^d^*M. graminicola*.

^1^Data were obtained from Zajc *et al.*^47^.

^2^Data were obtained from Lenassi *et al.*^45^.

^3^Data were obtained from Benito *et al.*^46^.

**Table 4:** BLASTX search results of *C. sphaerospermum* sequences with >50% identity match to allergens.

Biological function	Allergen ID	Accession number	Gene match (% identity)^a^
Aldehyde dehydrogenase	Cla h 10^b^/Alt a 10^b^	P40108/P42041	UM843_1101 (94/80),UM843_ 9748 (93/81),UM843_1184 (50/51), UM843_5878 (52/51), UM843_4714 (53/51)
Heat shock protein	Cla h HSP70	P40918	UM843_5039 (93)
Hydrophobin	Cla h HCh1	Q8NIN9	UM843_1201 (70), UM843_6061 (73), UM843_4115 (69), UM843_3639 (70)
Flavodoxin	Cla h 7^b^	P42059	UM843_8459 (82)
Mannitol dehydrogenase	Cla h 8^b^/Alt a 8^b^	P0C0Y5/P0C0Y4	UM843_6416 (94/74)
Acidic ribosomal protein P2	Cla h 5^b^	P42039	UM843_8958 (81)
Acidic ribosomal protein P1	Cla h 12^b^	P50344	UM843_3849 (76)
Enolase	Cla h 6^b^/Alt a 6^b^	P42040/Q9HDT3	UM843_9192 (95/91)
Nuclear transport factor	Cla h NTF2	Q8NJ52	UM843_8043 (94)
Vacuolar serine protease	Cla h 9^b^/Asp f 18^b^	AAX14379/P87184	UM843_8856 (91/70)
Ribosomal protein L3	Asp f 23^b^	Q8NKF4	UM843_6315 (85)
Fibrinogen binding protein	Asp f 2^b^	P79017	UM843_1014 (54), UM843_2103 (52)
Thioredoxin	Fus c 2^b^	Q8TFM8	UM843_3626 (55)
Disulfide-isomerase	Alt a 4^b^	Q00002	UM843_1562 (65)
Heat shock protein	Asp f 12^b^	P40292	UM843_1120 (85)
Alcohol dehydrogenase	Cand a 1^b^	P43067	UM843_1327 (61), UM843_9747 (62)
Aldolase	Cand a FPA	Q9URB4	UM843_7727 (68)

^a^For genes that have matches to two accession numbers, the percentage of identity showed matches to the first and second protein respectively.

^b^Allergens that are fully characterized and approved by the Allergen Nomenclature Sub-committee of the International Union of Immunological Societies (IUIS) (www.allergen.org).
