# Supplementary material for: Insight into different environmental niches adaptation and allergenicity from the Cladosporium sphaerospermum genome, a common human allergy-eliciting Dothideomycetes
Source: Sci Rep. 2016 May 31;6:27008. doi: 10.1038/srep27008 (PMC4886633; doi:10.1038/srep27008)
Supplement: Supplementary Dataset 2 [file srep27008-s2.doc]

**Insight into different environmental niches adaptation and allergenicity from the *Cladosporium sphaerospermum* genome, a common human allergy-eliciting Dothideomycetes**

Su Mei Yew^1^, Chai Ling Chan^1^, Yun Fong Ngeow^3^, Yue Fen Toh^1^, Shiang Ling Na^1^, Kok Wei Lee^2^, Chee-Choong Hoh^2^, Wai-Yan Yee^2^, Kee Peng Ng^1^, Chee Sian Kuan^1^*

^1^Department of Medical Microbiology, Faculty of Medicine, University of Malaya, Kuala Lumpur, Malaysia

^2^Codon Genomics SB, No. 26, Jalan Dutamas 7, Taman Dutamas, Balakong, 43200 Seri Kembangan, Selangor Darul Ehsan, Malaysia

^3^Department of Pre-Clinical Sciences, Faculty of Medicine and Health Sciences, Universiti Tunku Abdul Rahman, Bandar Sungai Long, 43000 Kajang, Selangor Darul Ehsan, Malaysia

*cs_sam85@yahoo.com.my


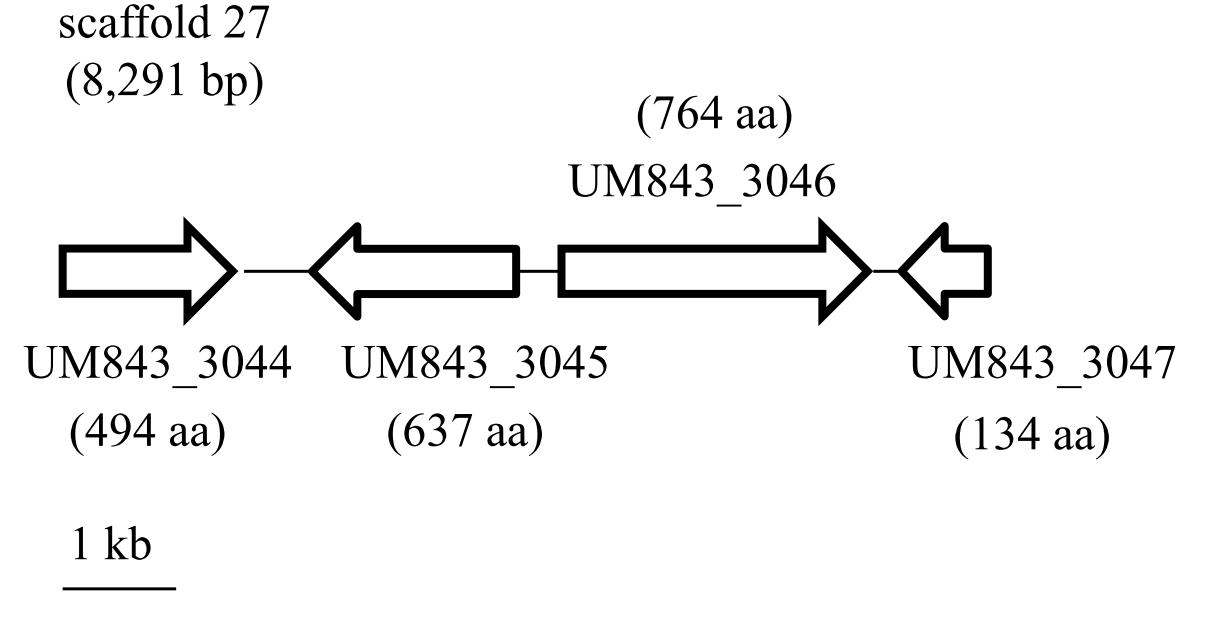


Fig S1. Putative gene organisation of mating type gene cluster in UM 843. The neighbouring genes of *Mat1-2* gene (UM843_3044) encompass the DNA lyase *Apn2* gene (UM578_3045), anaphase promoting complex subunit 5 gene (UM843_3046) and cytochrome C oxidase Vla *Cox13* gene (UM843_3047). The direction of transcription is indicated by the arrow for each gene.


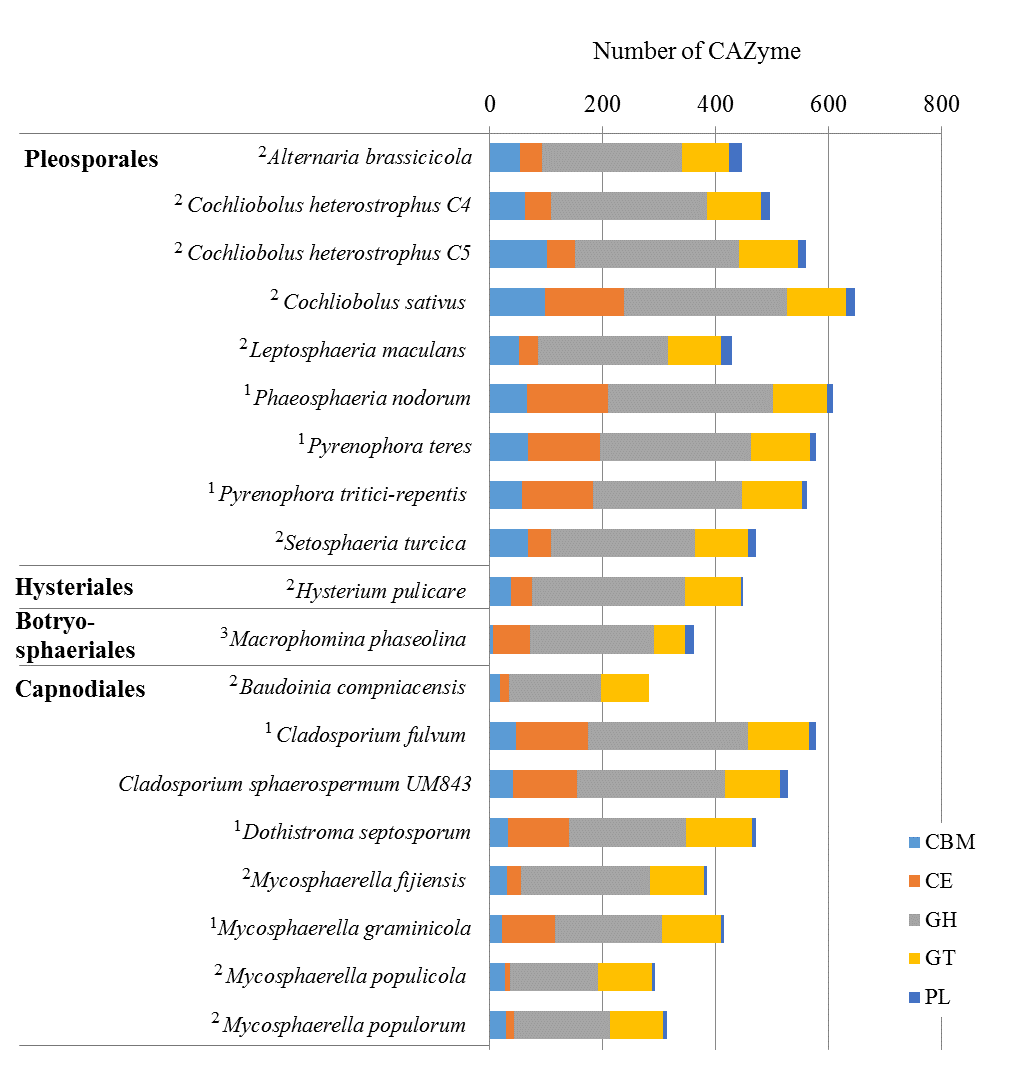


Fig. S2. Comparative analysis of *C. sphaerospermum* UM 843 CAZymes with other fungi in the class Dothideomycetes.

The candidate fungi chosen in this analysis consist of known plant pathogens (*Alternaria brassicicola*, *Pyrenophora tritici-repentis*, *P. teres*, *Cochliobolus heterostrophus* C4, *C. heterostrophus* C5, *C. sativus*, *Setosphaeria turtica*, *Leptosphaeria maculans*, *Phaeosphaeria nodorum*, *Macrophomina phaseolina*, *Cladosporium fulvum*, *Dothistroma septosporum*, *Mycosphaerella fijiensis*, *M. graminicola*, *M. populicola* and *M. populorum*) and saprophytes (*Hysterium pulicare* and *Baudoinia compniacensis*).

CBM, carbohydrate-binding modules; CE, carbohydrate esterases; GH, glycoside hydrolases; GT, glycosyltransferases; PL, polysaccharide lyases.

^1^ Data were obtained from Zhao *et al*. ^1^

^2^ Data were obtained from Ohm *et al*. ^2^

^3^ Data was obtained from Islam *et al*. ^3^


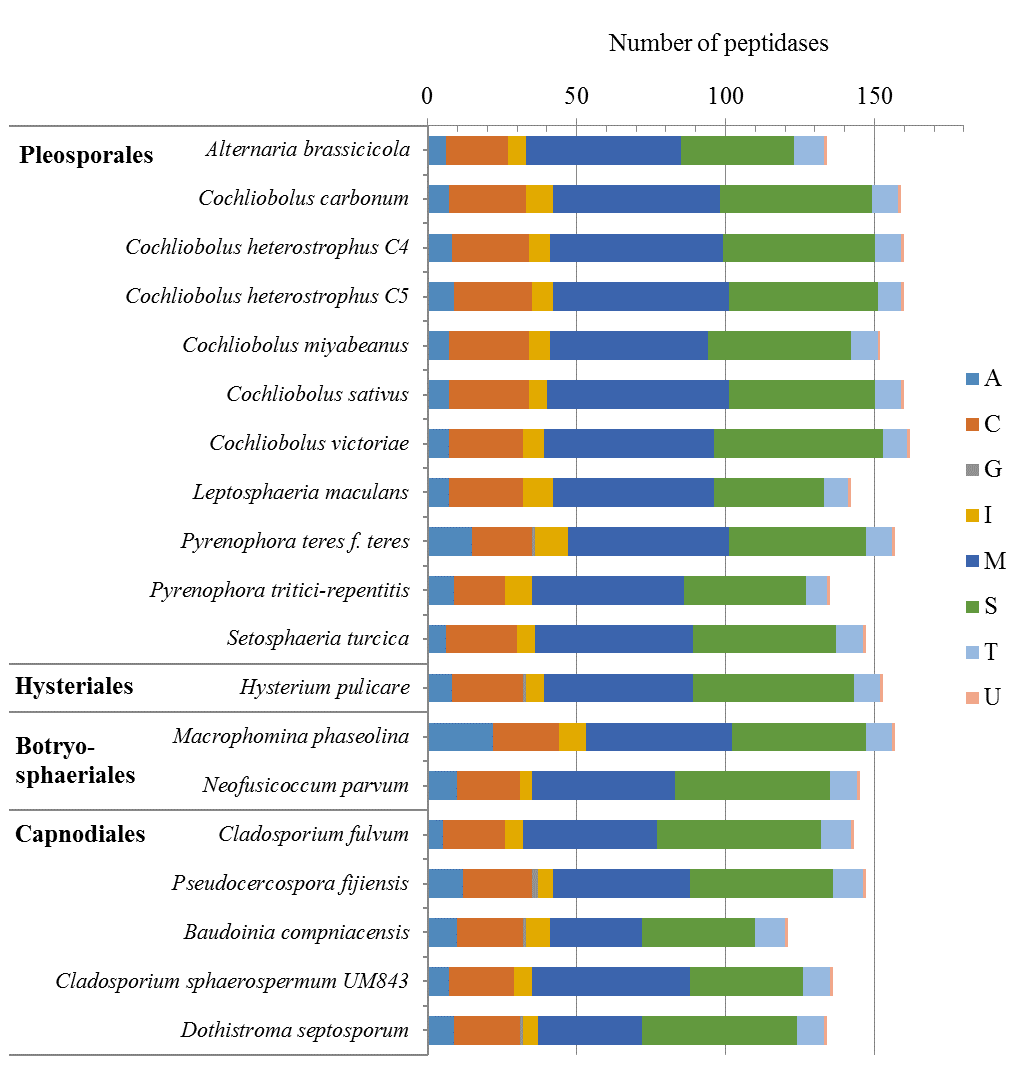


Fig. S3. Peptidases distribution in the class Dothideomycetes. Average amount of peptidases in *C. sphaerospermum* UM 843 compared to other members in Capnodiales.

A, aspartic; C, cysteine; G, glutamic; I, inhibitor; M,: metallo; S, serine; T, threonine; U, unknown.

UM843_2883 01 MTAVSLACAATLLIGSSIAIDPPRRPVAPTGAGNESLTFNNTVVSPQFRASTLSVDWLPD
ASPFU_DPPIV 1 ---MKWS--ILLLVGCAAAIDVPRQPYAPTGSGKKRLTFNETVVKRAISPSAISVEWIST
ASPOR_DPPIV 1 ---MKYSKLLLLLVSVVQALDVPRKPHAPTGEGSKRLTFNETVVKQAITPTSRSVQWLSG

UM843_2883 061 EEDGLVVYQADDGALIFENYATGENDTFVAADLIPE--DVYEYWIRSDQEKVLFSTNYTQ
ASPFU_DPPIV 56 SEDGDYVYQDQDGSLKIQSIVTNHTQTLVPADKVPE--DAYSYWIHPNLSSVLWATNYTK
ASPOR_DPPIV 58 AEDGSYVYAAEDGSLTIENIVTNESRTLIPADKIPTGKEAFNYWIHPDLSSVLWASNHTK

UM843_2883 0119 NYRHSYWSNYIVLDVASGEQTPLVDDQNGDIQYAQFAPTGDLIAFVRANNLYIKDVATGE
ASPFU_DPPIV 114 QYRHSYFADYFIQDVQSMKLRPLAPDQSGDIQYAQWSPTGDAIAFVRGNNVFVWT--NAS
ASPOR_DPPIV 118 QYRHSFFADYYVQDVESLKSVPLMPDQEGDIQYAQWSPVGNTIAFVRENDLYVWD--NGT

UM843_2883 0179 ISQITNDGGEDMFHGVPDWVYEEEIFGDRYTLWFSPDARFLSFLSFNETGVGTFTVQYFM
ASPFU_DPPIV 172 TSQITNDGGPDLFNGVPDWIYEEEILGDRFALWFSPDGAYLAFLRFNETGVPTFTVPYYM
ASPOR_DPPIV 176 VTRITDDGGPDMFHGVPDWIYEEEILGDRYALWFSPDGEYLAYLSFNETGVPTYTVQYYM

UM843_2883 0239 NDSDIAPEYSQNLDIRYPKVGTTNPTVSLTLLDLESMDLSPVSADAFPPEELIIGEVAWV
ASPFU_DPPIV 232 DNEEIAPPYPRELELRYPKVSQTNPTVELNLLELRTGERTPVPIDAFDAKELIIGEVAWL
ASPOR_DPPIV 236 DNQEIAPAYPWELKIRYPKVSQTNPTVTLSLLNIASKEVKQAPIDAFESTDLIIGEVAWL

UM843_2883 0299 TDTHESLIYRAYNRVQTQEKLVTVSVPSASTTVHRERDGSDGWLENLLNIQYVGQVRGNS
ASPFU_DPPIV 292 TGKHDVVAVKAFNRVQDRQKVVAVDVASLRSKTISERDGTDGWLDNLLSMAYIGPIGES-
ASPOR_DPPIV 296 TDTHTTVAAKAFNRVQDQQKVVAVDTASNKATVISDRDGTDGWLDNLLSMKYIGPIKPS-

UM843_2883 0359 TYSGSNTTYYLDLSDASGWAHIYLFPVNGPASNNITLTSGEWEVRSILSIDTQRQLVYYT
ASPFU_DPPIV 351 -----KEEYYIDISDQSGWAHLWLFPVAGG--EPIALTKGEWEVTNILSIDKPRQLVYFL
ASPOR_DPPIV 355 ----DKDAYYIDISDHSGWAHLYLFPVSGG--EPIPLTKGDWEVTSILSIDQERQLVYYL

UM843_2883 0419 STERHSTESHLYSVSFATLAKKALVDDSEAGWWSASFSSNNGYYLLNYGGPDVPYQELYA
ASPFU_DPPIV 404 STKHHSTERHLYSVSWKTKEITPLVDDTVPAVWSASFSSQGGYYILSYRGPDVPYQDLYA
ASPOR_DPPIV 409 STQHHSTERHLYSVSYSTFAVTPLVDDTVAAYWSASFSANSGYYILTYGGPDVPYQELYT

UM843_2883 0479 VNSSNPISTVTSNQALVDRIQDYNLPNTTWTQIGPVPDTEYMLNVKVTYPPNFDPSKKYP
ASPFU_DPPIV 464 INSTAPLRTITSNAAVLNALKEYTLPNITYFELAL-PS-GETLNVMQRLPVKFSPKKKYP
ASPOR_DPPIV 469 TNSTKPLRTITDNAKVLEQIKDYALPNITYFELPL-PS-GETLNVMQRLPPGFSPDKKYP

UM843_2883 0539 VIFTPYGGPNSQRVTKAYTTPNWNIYVAAEPELQYILYTIDNRGGAYQGREFRATVTEQL
ASPFU_DPPIV 522 VLFTPYGGPGAQEVSKPWQALDFKAYIASDPELEYITWTVDNRGTGYKGRAFRCQVASRL
ASPOR_DPPIV 527 ILFTPYGGPGAQEVTKRWQALNFKAYVASDSELEYVTWTVDNRGTGFKGRKFRSAVTRQL

 0•••••
UM843_2883 0599 GILEAKDQIWAADYLISQHDYIDADHVALWGWSFGGFLTAKVLETQGSDAGPFTLGLITA
ASPFU_DPPIV 582 GELEAADQVFAAQQA-AKLPYVDAQHIAIWGWSYGGYLTGKVIET---DSGAFSLGVQTA
ASPOR_DPPIV 587 GLLEAEDQIYAAQQA-ANIPWIDADHIGIWGWSFGGYLTSKVLEK---DSGAFTLGVITA

 0*
UM843_2883 0659 PVTDWRFYDSMYTERYMRTPATNAEGYEATAIRDTTGFKTVSGGFSIQHGLGDDNVHYQH
ASPFU_DPPIV 638 PVSDWRFYDSMYTERYMKTLESNAAGYNASAIRKVAGYKNVRGGVLIQHGTGDDNVHFQN
ASPOR_DPPIV 643 PVSDWRFYDSMYTERYMKTLSTNEEGYETSAVRKTDGFKNVEGGFLIQHGTGDDNVHFQN

 0*
UM843_2883 0719 TAALVDLLVGDGVTPEKMDWRVYTDSDHSIAYNGANVHLYKYLSKKLYDEKNREAGLVEQ
ASPFU_DPPIV 698 AAALVDTLVGAGVTPEKLQVQWFTDSDHGIRYHGGNVFLYRQLSKRLYEEKKRKEK-GEA
ASPOR_DPPIV 703 SAALVDLLMGDGVSPEKLHSQWFTDSDHGISYHGGGVFLYKQLARKLYQEKNRQTQ-VLM

UM843_2883 0779 HGWSKRGLVDFK
ASPFU_DPPIV 757 HQWSKKSVL---
ASPOR_DPPIV 762 HQWTKKDLEE--

**Fig. S4. Alignment of predicted DPP IV (UM843_2833) of *C. sphaerospermum* UM 843.** Alignment was carried out with DPP IV from *A. fumigatus* (ASPFU_DPPIV; AAC34310.1) and *A. oryzae* (ASPOR_DPPIV; Q2UH35). The consensus motif Gly-X-Ser-X-Gly is indicated with circle (•) and the catalytic triad Ser 631, Asp 711, His 746 are indicated with asterisk (*).

UM843_1649 1 MVAGQIAAAAALLLPLAAAITPEQMLSAPRYSAASSNPSGEWAVYTSTNYSFETQEAATV
ASPFU_DPPV 1 MGAFRWLSIAAA-ASTALALTPEQLITAPRRSEAIPDPSGKVAVFSTSQYSFETHKRTSW
ASPTN_DPPV 1 MAALRWLSAVVAVSTTVLAITPEQMLSAPRRGEAIPNPSGNVALFSASQYSFDTKEKSSS


UM843_1649 61 WKLLNIKTGDISDLPFADDVSEMVWVGNTNTSVLYINGTNDEIPGGVTLWTTDLAISPIV
ASPFU_DPPV 60 WSLLDLKTGQTKVLTNDSSVSEIVWLSD--DSILYVNSTNADIPGGVELWVTQAS-SFAK
ASPTN_DPPV 61 WNLLNLKTGDITLLTDDANVSEIVWLGGDDTSLLYINGTNAEIPGGVELWVSSVK-DFSK


UM843_1649 121 GTQVASLDAPFSGLKASNTPSGGINFVVNAKAYANNGSAYNEEFASTPATTGQLYDNVWV
ASPFU_DPPV 117 GYKAASLPASFSGLKAAKTKSGDIRFVAYGQSYP-NGTAYNEELATAPLSSARIYDSIYV
ASPTN_DPPV 120 GYKAASLGASFSGLKAVRTRSGDIKFAVNAQSYA-NGTAYNEELATKYASTARIYDSIYV


UM843_1649 181 RHWDTYVTQERFSVFGGSLAQSNS--SLSLSGNMTNLLLGIDAPITRPETPYQPFGGSSD
ASPFU_DPPV 176 RHWDYWLSTTFNAVFSGTLKKGHGKNGYSLDGELK----NLVSPVKNAESPYPPFGGASD
ASPTN_DPPV 179 RHWDTYLTTTFNAVFAGTLKKG--KHQYASAGPLK----NLVAPVKNAESPYPPFGDSTD


UM843_1649 239 YDLSPDGSQVVFMTKAPELPKANFTASYLYIVPHDGSSVAERLNGPNST-APEEAKGASA
ASPFU_DPPV 232 YDLSPDGKWVAFKSKAPELPKANFTTSYIYLVPHDASETARPINGPDSPGTPKGIKGDSS
ASPTN_DPPV 233 YDVSPDGKWVAFKSKAPDVPRANYTTAYIYLAPHDGSSTATPINGPDSPGTPEGVQGDAN


UM843_1649 298 NPVWSPDGKRIAYQQMDGINYESDRAKLYIADVASGE-ITLLAADWDVSVGSIKWSHDCD
ASPFU_DPPV 292 SPVFSPNGDKLAYFQMRDETYESDRALLYVYSLGSKKTIPSVAGDWDRSPDSVKWTPDGK
ASPTN_DPPV 293 YPVFSPDSRHLAYFQMAHKSYESDRRVLYVYTLGSKTTTPAVAGDWNRSPDSAKWLD-NK


UM843_1649 357 ELYVTGDYIGSTRLFI-VPADAAADYKPENVTDSTTVVDFNVLPNGDSLISSSAVWSSRM
ASPFU_DPPV 352 TLIVGSEDLGRTRLFS-LPANAKDDYKPKNFTDGGSVSAYYFLPDSSLLVTGSALWTNWN
ASPTN_DPPV 352 HLILGSEDHARVRLFGPVPIDADDDFKPQNFTDGGAVSSYHVLPDKTVLVTGTAIWTSWN


UM843_1649 416 IYTVTPDSKT-TYLYKAQDVDAELAGLGPDDLSFEWYTGTLGDQQQMLVIYPEGFTKNKT
ASPFU_DPPV 411 VYTAKPEKGVIKKIASANEIDPELKGLGPSDISEFYFQGNFTDI-HAWVIYPENFDKSKK
ASPTN_DPPV 412 VYTASPKKGVIKTIASANKIDPGLAGLGPEDISEFYYDGNWTKI-QSWIIYPENFDSSKK


UM843_1649 475 YDLAFIVHGGPQGLHANSWSTRWNFKVWADQGYVVVAPNPTGSTSYGQALTDRIQGRWST
ASPFU_DPPV 470 YPLIFFIHGGPQGNWADGWSTRWNPKAWADQGYVVVAPNPTGSTGFGQALTTAIQNNWGG
ASPTN_DPPV 471 YPLFFYIHGGPQSATPDSWSTRWNAKVFADQGYVVVAPNPTGSTGFGQELTDAIANNWGG

 0•••••
UM843_1649 535 WPYEDLVNAFAHVRDTMPFVNTSNAIEAGASYGGYMTNWIQGHDLGREFKALVAHDGVTT
ASPFU_DPPV 530 APYDDLVKCWEYVHENLDYVDTDHGVAAGASYGGFMINWIQGSPLGRKFKALVSHDGTFV
ASPTN_DPPV 531 APYEDLVKAWEYVDKNLPYVDTENGVAAGASYGGFMINWIQGSDLGRKFKALVCHDGTFV

 0*
UM843_1649 595 TFSDFGTEELFFMLHDQNGTLWDNRETYAMNDPFTHA--RNFSTPQLIIHNDLDYRLPVS
ASPFU_DPPV 590 ADAKVSTEELWFMQREFNGTFWDARDNYRRWDPSAPERILQFATPMLVIHSDKDYRLPVA
ASPTN_DPPV 591 ADAKISTEELWFIEHDFNGTFWGARDNYRRWDPSAPERILQFSTPQLVIHSDQDYRLPVA

 o *
UM843_1649 653 EGIAMFNALQLLGVPSRFLHFPDENHWVLNRENSLLWHHTIFDWINYYTGKTESLSDEGV
ASPFU_DPPV 650 EGLSLFNVLQERGVPSRFLNFPDENHWVVNPENSLVWHQQALGWINKYSGVEKSNPNAVS
ASPTN_DPPV 651 EGLAMFNVLQERGVPSRFLNFPDENHWVLKQENSLVWHQQMLGWLNRYSGIEEANPDAVS


UM843_1649 713 ITQ----------
ASPFU_DPPV 710 LEDTVVPVVNYN-
ASPTN_DPPV 711 LDDTIIPVVNYNP

**Fig. S5. Alignment of predicted DPP V (UM843_1649) of *C. sphaerospermum* UM 843.** Alignment was carried out with DPP V from *A. fumigatus* (DPPV_Afumi; AAB67282) and *A. terreus* (DPP5_ASPTN; Q0C8V9.1). The consensus motif Gly-X-Ser-X-Gly is indicated with circle (•) and the catalytic triad Ser 565, Asp 646, His 678 are indicated with asterisk (*).

**
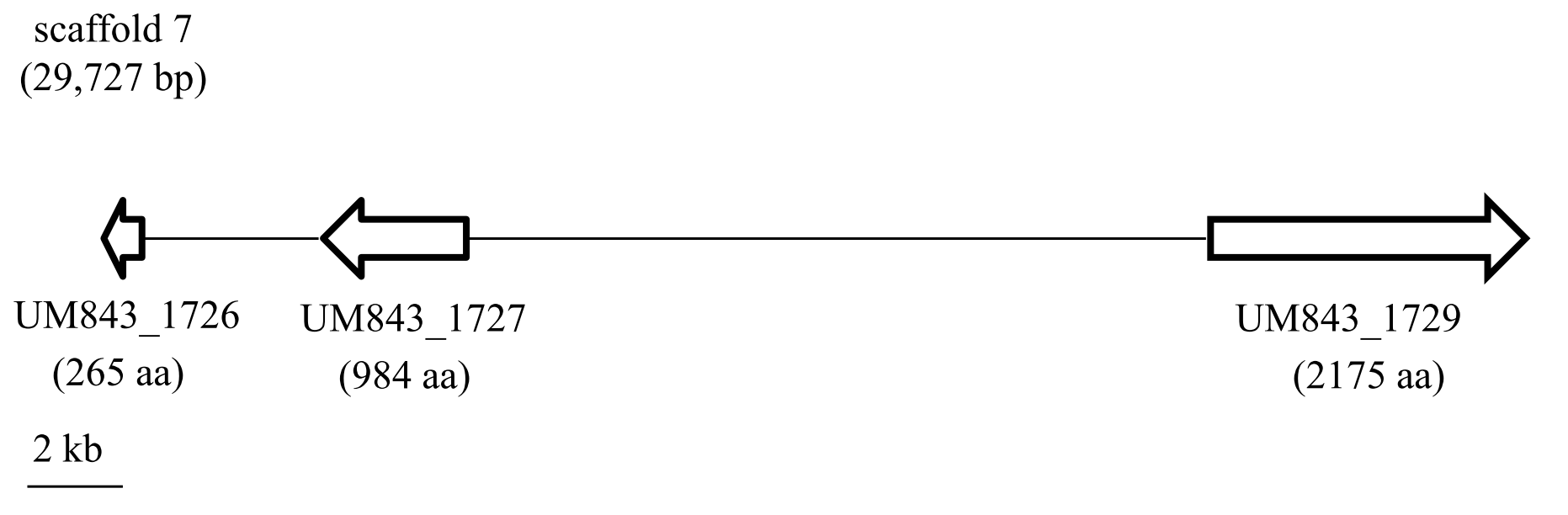
**

**Fig. S6. Putative melanin biosynthesis cluster in UM 843.** The genes are predicted to encode tetrahydroxynaphthalene reductase (UM843_1726), transcription factor Cmr1 (UM843_1727) and PKS (UM843_1729). The direction of transcription is indicated by the arrow for each gene.

**
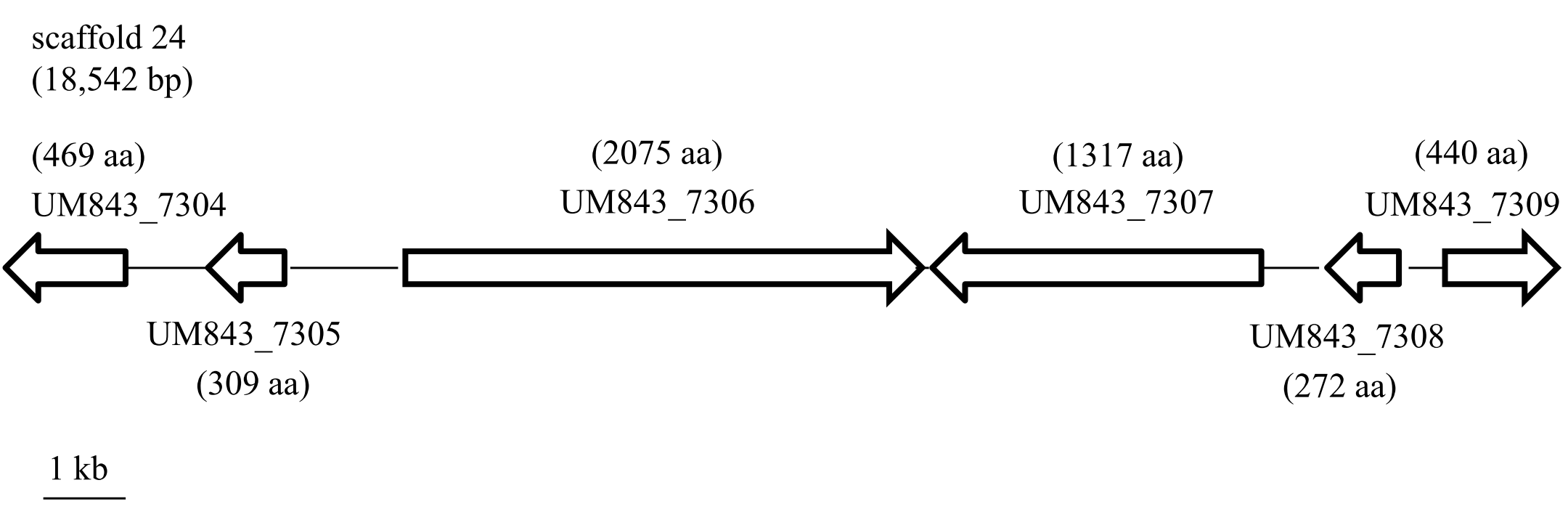
**

**Fig. S7. Putative fusarinine-type siderophore biosynthesis cluster in UM 843.** The genes are predicted to encode L-ornithine-N^5^-monooxygenase (UM843_7304), esterase (UM843_7305), NRPS SidD(UM843_7306), transporter SitT (UM843_7307), carnitinyl-CoA dehydratase (UM843_7308), acetyltransferase SidF (UM843_7309). The direction of transcription is indicated by the arrow for each gene.

**
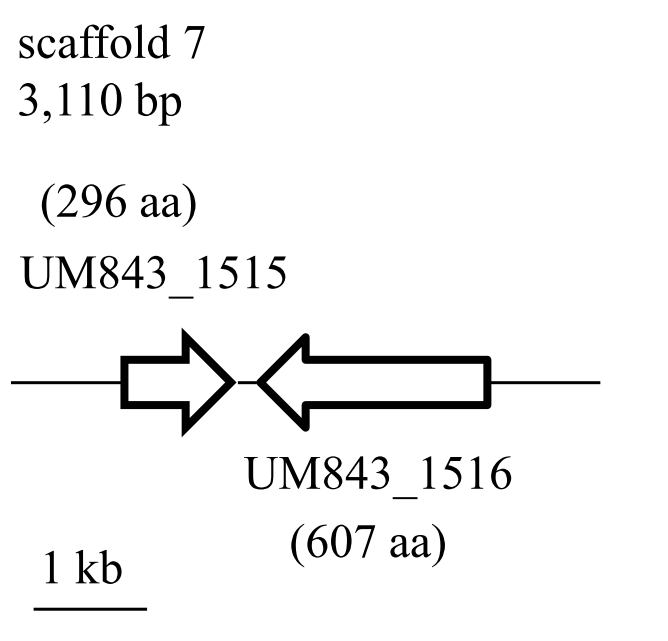
**

**Fig. S8. Putative cluster of genes involved in transportation of triacetyl-fusarinine C (TAFC).** The genes are predicted to encode siderophore esterase EstB (UM843_1515) and siderophore transporter MirB (UM843_1516). The direction of transcription is indicated by the arrow for each gene.

**
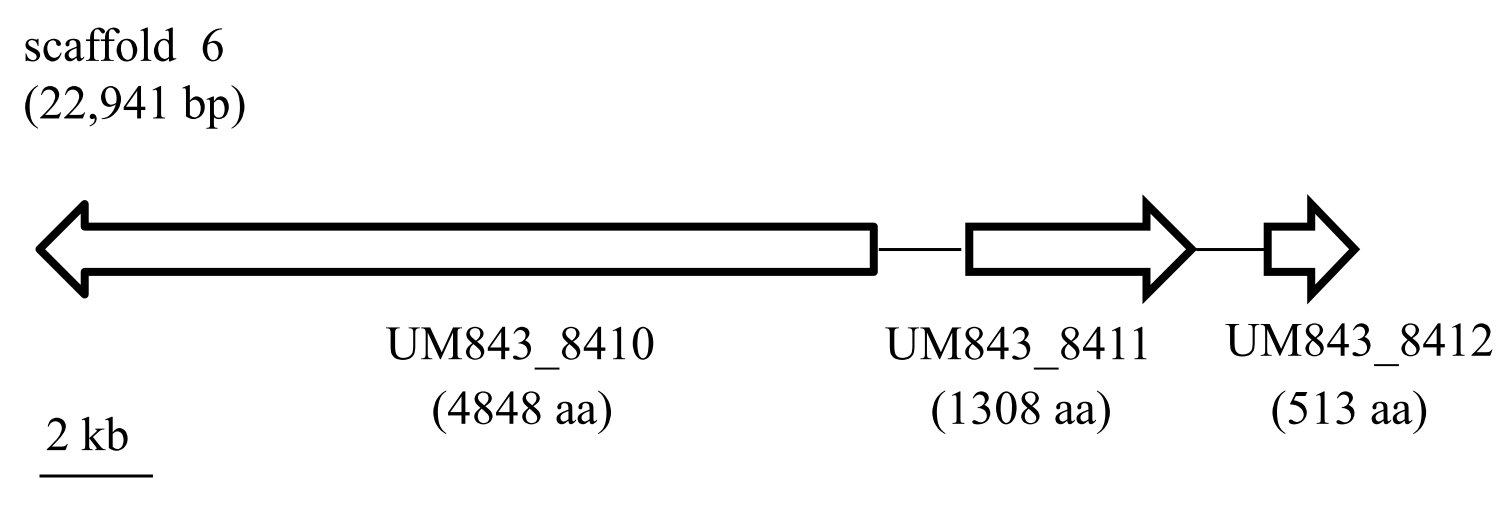
**

**Fig. S9. Putative gene cluster involved in ferrichrome-type siderophore biosynthesis.** The genes are predicted to encode ferrichrome-type NRPS (UM843_8410), ABC transporter (UM843_8411) and L-ornithine-N^5^-monooxygenase (UM843_8412). The direction of transcription is indicated by the arrow for each gene.

**
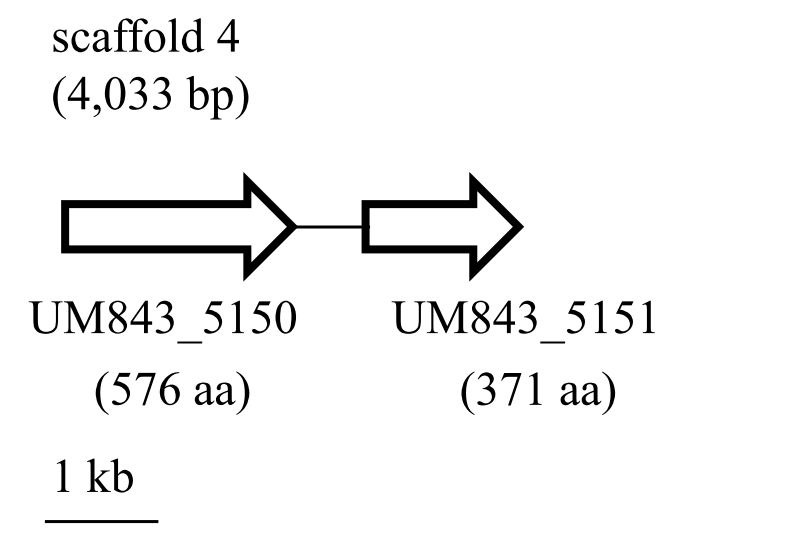
**

**Fig. S10. Putative reductive iron assimilation (RIA) system cluster in UM 843.** The genes are predicted to encode ferroxidase FetC (UM843_5150) and iron permease Ftr1 (UM843_5151). The direction of transcription is indicated by the arrow for each gene.


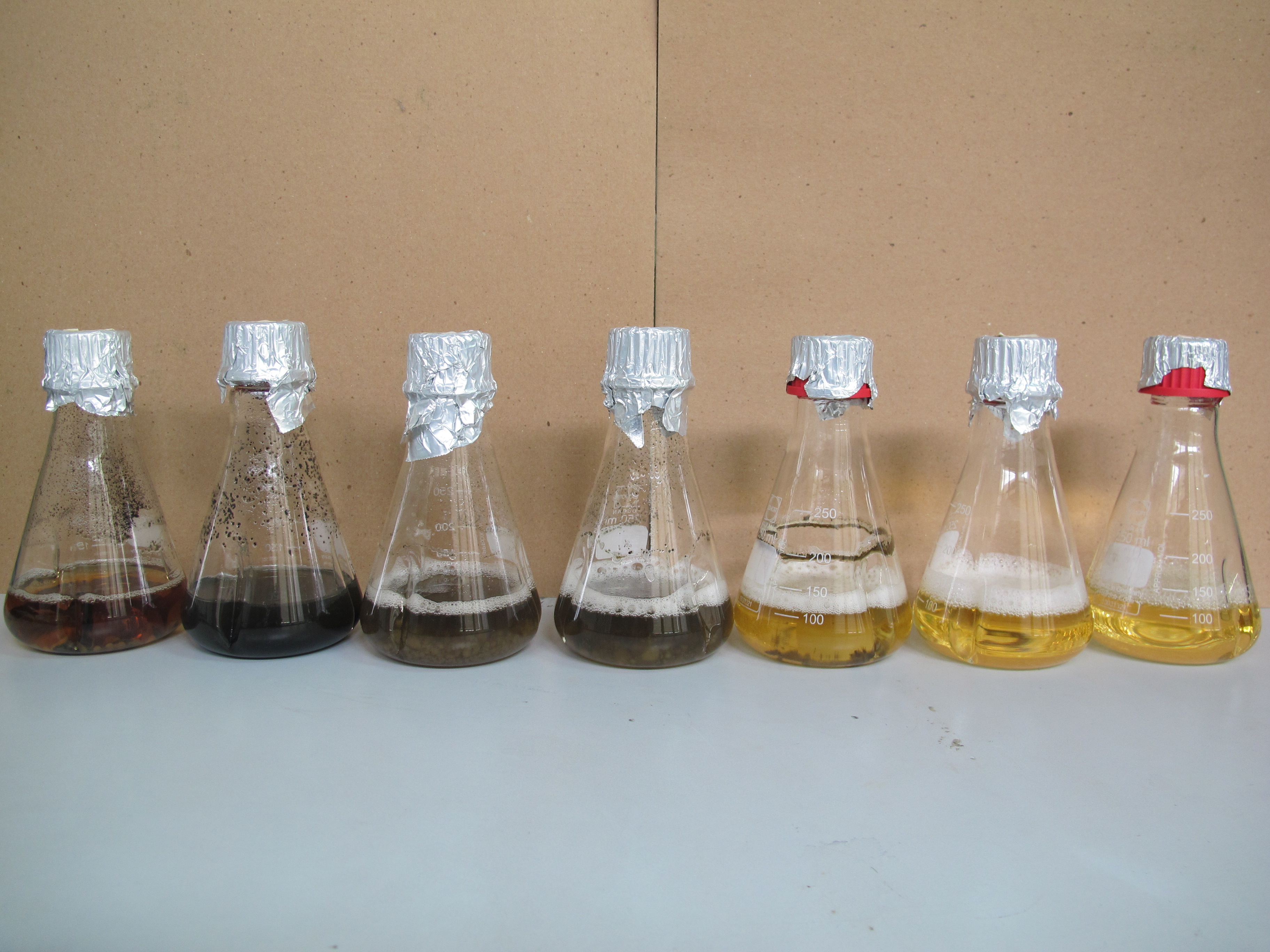


**a)**

**b)**

**c)**

**d)**

**e)**

**f))**

**g)**

**Fig. S11. Growth of *C. sphaerospermum* UM 843 in Sabouraud Dextrose Broth (SDB) supplemented with various percentage (w/v) of NaCl.** a) 0%, b) 5%, c) 10%, d) 15%, e) 20%, f) 25%, g) 30%

********* ******

WiGpd1_CBW47554 01 -------------------------------MVKESVAVIGSGNWGSVAARMAGQSCLEK
ScGpd1_CAA54189 01 MSAAADRLNLTSGHLNAGRKRSSSSVSLKAAEKPFKVTVIGSGNWGTTIAKVVAENCKGY
UM843_9164 01 -----------------MASNSNNNAGLGVHSKKHRVAIVGSGNWGTTIAKVVGENVQEN
HwGpd1A_AEM44788 1 ------------------------MASLSAHSQKHKVCVVGSGNWGTTIAKVVAENCKEH
HwGpd1B_AEM44789 1 ------------------------MAPLSTHSQKHKVCVVGSGNWGTTIAKVVAENCKEH


WiGpd1_CBW47554 030 PDLFERDVPMWVFEEQVD-----------------GRNLTDIINEKHENVKYLPGAKFPE
ScGpd1_CAA54189 061 PEVFAPIVQMWVFEEEIN-----------------GEKLTEIINTRHQNVKYLPGITLPD
UM843_9164 044 PDLFEQEVRMWVFEEEYSIPKNSKHYDAGSSLCTKPHKLTELINGLHENVKYLPGIALPQ
HwGpd1A_AEM44788 37 NDIFEEEVQMWVFEEQYQIPETSKHYDASNELTSKPQKLTSLINTFHENVKYLPNIALPH
HwGpd1B_AEM44789 37 NDIFEEEVQMWVFEEQYQIPETSKHYDASNELTSKPQKLTSLINTFHENVKYLPNIALPH


WiGpd1_CBW47554 073 NVIANPDLIDTVEDATLLIIVIPHQFLPKTLNTLKGHINPKARAVSLIKGVEVVGDNINI
ScGpd1_CAA54189 104 NLVANPDLIDSVKDVDIIVFNIPHQFLPRICSQLKGHVDSHVRAISCLKGFEVGAKGVQL
UM843_9164 104 NIVAHPDLATAVQDATILVFNLPHQFIAKTCQSIVGKIVPYARGISCIKGVAVNDSGCEL
HwGpd1A_AEM44788 97 NIVANPDLLDAVKGATILVFNLPHQFIAKTCDTLRGNIVPFARGISCVKGVDVSESGCEL
HwGpd1B_AEM44789 97 NIVANPDLLDAVKGATILVFNLPHQFIAKTCDTLRGNIVPFARGISCVKGVDVSESGCEL


WiGpd1_CBW47554 133 FAKVIERELGIRCSALSGANIANEIALEKFSETSIGYADRKD------------------
ScGpd1_CAA54189 164 LSSYITEELGIQCGALSGANIATEVAQEHWSETTVAYHIPKDFRG---------------
UM843_9164 0164 FSESIGRELGIYCGALSGANIANEVAQEKWSETTVAYDPPKMDSKHPSPVGTPH------
HwGpd1A_AEM44788 157 FSESIGEKLGIYCGALSGANIATEVALEKWSETTVAYDPPSMDSKHPTPAATPGGTPGGS
HwGpd1B_AEM44789 157 FSESIGEKLGIYCGALSGANIATEVALEKWSETTVAYDPPSMDSKHPTPAATPGGTPGGS


WiGpd1_CBW47554 0175 ---------------------------------------GEIFQKLFDRPYFRVSIVEDV
ScGpd1_CAA54189 0209 ---------------------------------EGKDVDHKVLKALFHRPYFHVSVIEDV
UM843_9164 0218 --ASSLDLTQLNGAEPESKKLKTSGPKLQPLPSEFPPLHHANMKKLFHRPYFHVRMVHDV
HwGpd1A_AEM44788 217 PKASHVDLTNLVAESI--KNASGKIQHLKSLPSEYPALHARNMKKLFHRPYFHVRIVSDV
HwGpd1B_AEM44789 217 PKASHVDLTNLVAESI--KNASGKIQHLKSLPSEYPALHPRNMKKLFHRPYFHVRIVSDV


WiGpd1_CBW47554 0196 EGVSLCGAMKNIVTIACGFNDGMNWGDNTKAAVMRVGLLEMKRFCQDFFPSC-KDATFVE
ScGpd1_CAA54189 0236 AGISICGALKNVVALGCGFVEGLGWGNNASAAIQRVGLGEIIRFGQMFFPES-REETYYQ
UM843_9164 0276 AGVSLGGALKNVVALAAGWVDGLGWGDNAKAAVMRVGIMEEVRFGKAFFAASTRTETFTE
HwGpd1A_AEM44788 275 AGVSLGGALKNVVALGAGWVDGLGWGDNAKAAVMRVGIMEEVRFGKMFFADTVRAESFTE
HwGpd1B_AEM44789 275 AGVSLGGALKNVVALGAGWVDGLGWGDNAKAAVMRVGIMEEVRFGKMFFGDTVRAESFTE


WiGpd1_CBW47554 0255 ESAGVADLITTCLGGRNRKVAAAHAETGKSFEQLEQEILGGQRLQGTGTAKEIHEFLSHK
ScGpd1_CAA54189 0295 ESAGVADLITTCAGGRNVKVARLMATSGKDAWECEKELLNGQSAQGLITCKEVHEWLETC
UM843_9164 0336 ESCGVADLITSCSGGRNFRCAKMSVAEGKSIDEIEARELNGQKLQGTSTAHEVYAFLKKE
HwGpd1A_AEM44788 335 ESCGVADMITSCGSGRNFRCAKMSAAEGKPISEIEKRELNGQQLQGTGTAYEVNAFLKMQ
HwGpd1B_AEM44789 335 ESCGVADMITSCGSGRNFRCAKMSAAEGKPIAEIEKRELNGQQLQGTGTAYEVNAFLKKE


WiGpd1_CBW47554 0315 GKVDNYPLFKTVYEIAFENKPAKELTSRL--------------
ScGpd1_CAA54189 0355 GSVEDFPLFEAVYQIVYNNYPMKNLPDMIEELDLHED------
UM843_9164 0396 GREKEFPLFTAVRNILEGVNEPKDIPDLIEEGQREME------
HwGpd1A_AEM44788 395 GKEKEFPLFTAVYNILEGKYQAKDIPDLIEQKD----------
HwGpd1B_AEM44789 395 GKEKDFPLFTAVYLILEGKYQAKDIPDLIEQKDEQKLISEEDL


**Fig. S12. Alignment of NAD-dependent glycerol-3-phosphate dehydrgenase (Gdp) of UM 843 (UM843_9164).** Alignment was carried out with Gdp of *Wallemia ichthyophaga* (WiGpd1; CBW47554), *Saccharomyces cerevisiae* (ScGpd1; CAA54189) and *Hortaea werneckii* (HwGpd1A; AEM44788 and HwGpd1B; AEM47789). The peroxisomal targeting signal type 2 (PTS2) in indicated in box and the consensus NAD(P)/NAD(P)H binding site GXGXXG is indicated in asterisk (*). The Gdp N-terminus domain (PF01210) and C-terminus domain (PF07479) are shown in solid and dash line, respectively.


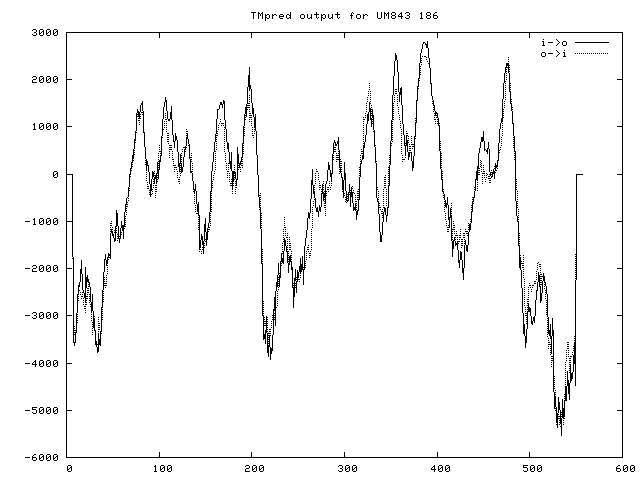

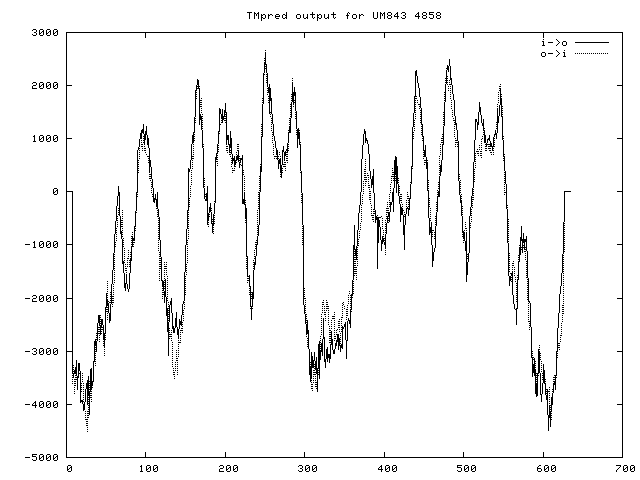


a)

b)

c)

d)


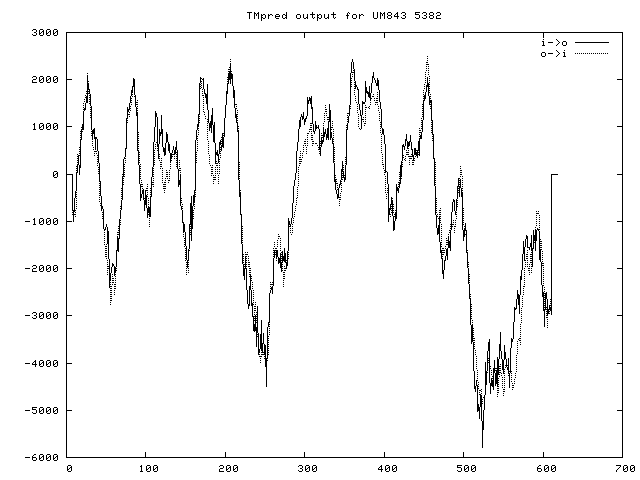

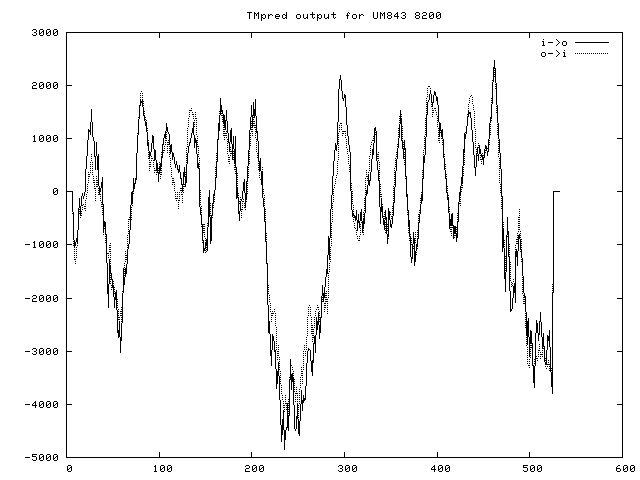


e)

f)


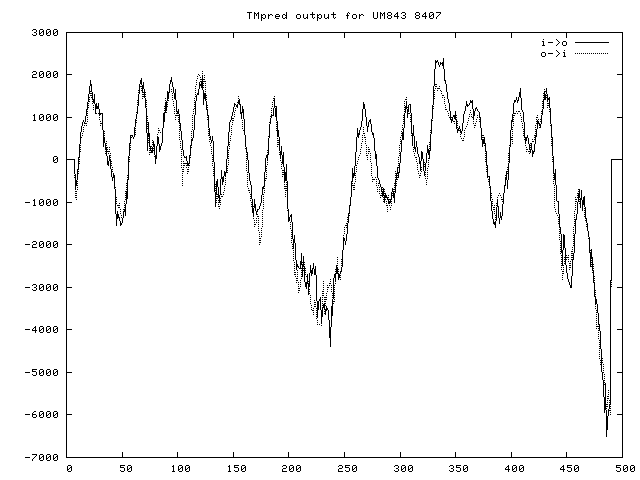

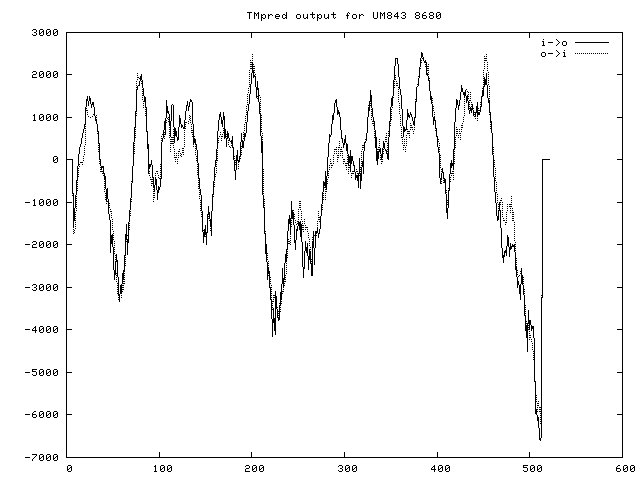


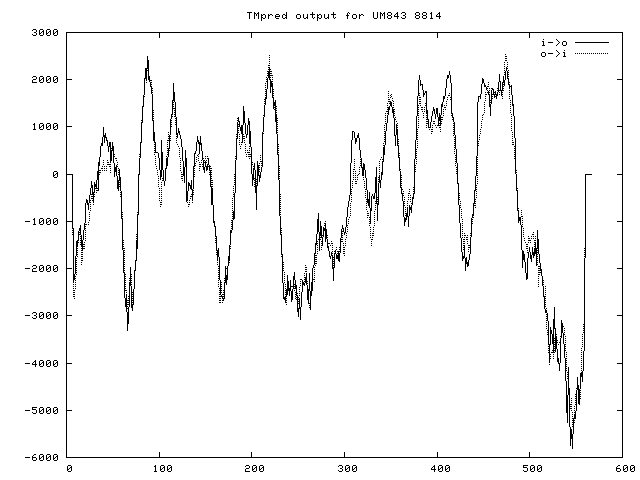


g)

**Fig. S13. TMpred output of putative glycerol/H^+^ symporter (*slt1*) in UM 843.** a) UM843_186, b) UM843_4858, c) UM843_5382, d) UM843_8200, e) UM843_8407, f) UM843_8680 and g) UM843_8814. The putative genes have twelve transmembrane domains.


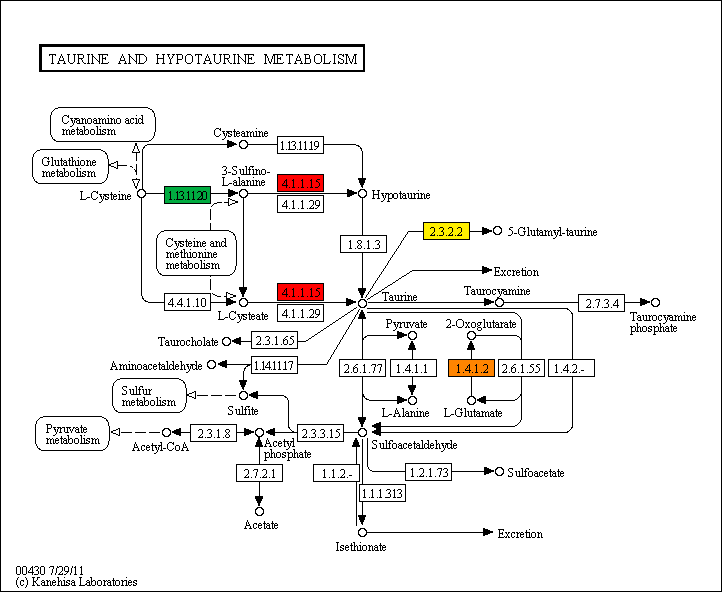


**Fig. S14. Taurine biosynthesis pathway of UM 843 predicted via KEGG pathway map.**

ScHog1_P32485 oo01 MTTNEEFIRTQIFGTVFEITNRYNDLNPVGMGAFGLVCSATDTLTSQPVAIKKIMKPFST
AfSakA_XM_747571 1 ---MAEFVRAQIFGTTFEITSRYTDLQPVGMGAFGLVCSARDQLTGQPVAVKKIMKPFST
UM843_5411 01 ---MAEFVRAQIFGTTFEITSRYSDLQPVGMGAFGLVCSAKDQLTGSAVAVKKIMKPFST
HwHog1_AAM64214 01 ---MAEFVRAQIFGTTFEITSRYTDLQPVGMGAFGLVCSAKDQLTSQAVAVKKIMKPFST


ScHog1_P32485 o0 61 AVLAKRTYRELKLLKHLRHENLICLQDIFLSPLEDIYFVTELQGTDLHRLLQTRPLEKQF
AfSakA_XM_747571 58 PVLSKRTYRELKLLKHLRHENIISLSDIFISPLEDIYFVTELLGTDLHRLLTSRPLEKQF
UM843_5411 058 PVLSKRTYRELKLLKHLRHENVISLSDIFISPLEDMYTVTELLGTDLHRLLTSRPLEKQF
HwHog1_AAM64214 058 PVLSKRTYRELKLLKHLRHENIICLSDIFISPLEDMYVVTELLGTDLHRLLTSRPLEKQF

 0***
ScHog1_P32485 o0 121 VQYFLYQILRGLKYVHSAGVIHRDLKPSNILINENCDLKICDFGLARIQDPQMTGYVSTR
AfSakA_XM_747571 118 IQYFLYQILRGLKYVHSAGVVHRDLKPSNILINENCDLKICDFGLARIQDPQMTGYVSTR
UM843_5411 0118 IQYFLYQILRGLKYVHSAGVVHRDLKPSNILVNENCDLKICDFGLARIQDPQMTGYVSTR
HwHog1_AAM64214 0118 IQYFLYQILRGLKYVHSAGVVHRDLKPSNILINENCDLKICDFGLARIQDPQMTGYVSTR


ScHog1_P32485 o0 181 YYRAPEIMLTWQKYDVEVDIWSAGCIFAEMIEGKPLFPGKDHVHQFSIITDLLGSPPKDV
AfSakA_XM_747571 178 YYRAPEIMLTWQKYDVEVDIWSAGCIFAEMLEGKPLFPGKDHVNQFSIITELLGTPPDDV
UM843_5411 0178 YYRAPEIMLTWQKYDVEVDIWSAGCIFAEMLEGKPLFPGKDHVNQFSIITELLGTPPDDV
HwHog1_AAM64214 0178 YYRAPEIMLTWQKYDVEVDIWSAGCIFAEMLEGKPLFPGKDHVNQFSIITELLGTPPDDV


ScHog1_P32485 o0 241 INTICSENTLKFVTSLPHRDPIPFSERFKTVEPDAVDLLEKMLVFDPKKRITAADALAHP
AfSakA_XM_747571 238 IQTICSENTLRFVKSLPKRERQPLANKFKNADPEAVDLLERMLVFDPKKRIRAGEALAHE
UM843_5411 0238 ISTICSENTLRFVQSLPKRERQPLKNKFKNADPQAIELLEKMLVFDPRTRIKAGEALADP
HwHog1_AAM64214 0238 IATICSENTLRFVQSLPKRERQPLKNKFKNADPQAIELLERMLVFDPRKRVKAGEALADP

 ♦ 0♦♦
ScHog1_P32485 o0 301 YSAPYHDPTDEPVADAKFDWHFNDADLPVDTWRVMMYSEILDFHKIGGSDGQIDISATFD
AfSakA_XM_747571 298 YLSPYHDPTDEPEAEEKFDWSFNDADLPVDTWKIMMYSEILDFHNIDQGNDAGQVLM--E
UM843_5411 0298 YLAPYHDPTDEPVAEEKFDWSFNDADLPVDTWKIMMYSEILDYHNVDTNGDPASEAH---
HwHog1_AAM64214 0298 YLAPYHDPTDEPEAQEKFDWSFNDADLPVDTWKIMMYSEILDFHNVDANAEQAAHNN---


ScHog1_P32485o0 361 DQVAAATAAAAQAQAQAQAQVQLNMAAHSHNGAGTTGNDHSDIAGGNKVSDHVAANDTIT
AfSakA_XM_747571 356 GGVAQAQQ---------------NYA----------------------------------
UM843_5411 0355 GEIPRQV-----------------------------------------------------
HwHog1_AAM64214 355 DTVAG-------------------------------------------------------


ScHog1_P32485 421 DYGNQAIQYANEFQQ
AfSakA_XM_747571 ---------------
UM843_5411 ---------------
HwHog1_AAM64214 ---------------

**Fig. S15. Alignment of high osmolarity glycerol (Hog1) of UM 843 (UM843_5411).** Alignment was carried out with *Hortaea werneckii* Hog1 (HwHog1; AAM64214), Saccharomyces cerevisiae Hog1 (ScHog1; P32485) and Aspergillus fumigatus SakA (AfSakA; XM_747571). The activation loop and common docking domain are indicated in the first and second box, respectively. The conversed phosphorylation Lip is indicated in line while the asterisk (*) indicates the TYG phosphorylation site motif. The YHDP[T/S]DEP motif is indicated in line in the second box with rhombus (♦)indicating the negatively charged amino acid residues.


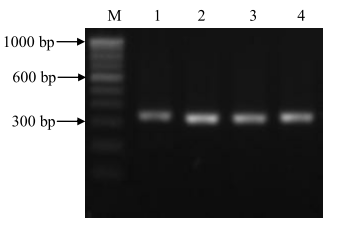


**Fig. S16. PCR amplification of putative hydrophobins ORF.** M: 100 bp DNA Ladder (i-DNA Biotechnology), Lane 1: UM843_6061, Lane 2: UM843_4115, Lane 3: UM843_3639, Lane 4: UM843_1201.


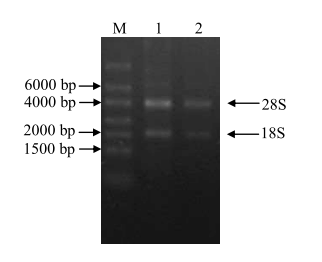


**Fig. S17. Agarose gel electrophoresis of extracted total RNA.** Lane M: High range riboRuler^TM^ RNA ladder and Lane 1-2: total RNA extracted from *C. sphaerospermum* UM 843. One microgram of extracted total RNA sample was loaded into each lane.

a1201_g 01 --------------ATGGCCTTCATCAAGACTCTCCTCATCGCCTCCGTGGCCGCCCTTG
b1201_t 01 --------------ATGGCCTTCATCAAGACTCTCCTCATCGCCTCCGTGGCCGCCCTTG
c1201_9A 1 ACCCAATCAACACAATGGCCTTCATCAAGACTCTCCTCATCGCCTCCGTGGCCGCCCTTG

a1201_g 047 CCTACGCCGCCCCCCAGGGCGCCTCTGACGGCAACACCAAGGTCTCCGCCTCCAAGGACT
b1201_t 047 CCTACGCCGCCCCCCAGGGCGCCTCTGACGGCAACACCAAGGTCTCCGCCTCCAAGGACT
c1201_9A 61 CCTACGCCGCCCCCCAGGGCGCCTCTGACGGCAACACCAAGGTCTCCGCCTCCAAGGACT

a1201_g 0107 CCCAGGAGGCTGCCTGCGGCAACGGCCAGAAGCTCGCCTGCTGCAACAGCGGCGAGGACC
b1201_t 0107 CCCAGGAGGCTGCCTGCGGCAACGGCCAGAAGCTCGCCTGCTGCAACAGCGGCGAGGACC
c1201_9A 121 CCCAGGAGGCTGCCTGCGGCAACGGCCAGAAGCTCGCCTGCTGCAACAGCGGCGAGGACC


a1201_g 0167 TCATCGGCGCCAACTGCCTGAACGTCCCCATCCGTACGTTTCCACGATTCCTCCAAACAT
b1201_t 0167 TCATCGGCGCCAACTGCCTGAACGTCCCCATCC---------------------------
c1201_9A 181 TCATCGGCGCCAACTGCCTGAACGTCCCCATCC---------------------------


a1201_g 0227 TTTATCGCTCCTTATCACTCCTTTACTGACACTCAAACACAGTGGCCGTCCCCGTCCAGC
b1201_t 0200 ------------------------------------------TGGCCGTCCCCGTCCAGC
c1201_9A 214 ------------------------------------------TGGCCGTCCCCGTCCAGC


a1201_g 0287 AGGCCTGCGGCTCCAACGTCGCCGCGTGCTGCAAGACCGGCGATGCCTCCGGCAACCTCA
b1201_t 0218 AGGCCTGCGGCTCCAACGTCGCCGCGTGCTGCAAGACCGGCGATGCCTCCGGCAACCTCA
c1201_9A 232 AGGCCTGCGGCTCCAACGTCGCCGCGTGCTGCAAGACCGGCGATGCCTCCGGCAACCTCA


a1201_g 0347 TCAACCTCGAGCTCAACTGCCTGTCCCTCCCCCTCTAA
b1201_t 0278 TCAACCTCGAGCTCAACTGCCTGTCCCTCCCCCTCTAA
c1201_9A 292 TCAACCTCGAGCTCAACTGCCTGTCCCTCCCCCTCTAA

**Fig. S18. Alignment of hydrophobin cDNA sequence (1201_9A) with the predicted DNA sequence (1201_g) and putative coding DNA sequence (1201_t) of *C. sphaerospermum* UM 843.** The start codon is in box and the intron region shaded in yellow. The intron size is 69 bp (position 200-268).

a3639_g 01 --------------------------ATGGCCTTCATGAAGACTCTCCTCATCGCCTCCG
b3639_t 01 --------------------------ATGGCCTTCATGAAGACTCTCCTCATCGCCTCCG
c3639_9A 1 --------------AAAACCAACAAAATGGCCTTCATGAAGACTCTCCTCATCGCCTCCG


a3639_g 035 TGGCTGCTGTTGCCTACGCCCAGGGCGGCTCTGACGGCGCCAAGAAGATCTCCGTCGACA
b3639_t 035 TGGCTGCTGTTGCCTACGCCCAGGGCGGCTCTGACGGCGCCAAGAAGATCTCCGTCGACA
c3639_9A 47 TGGCTGCTGTTGCCTACGCCCAGGGCGGCTCTGACGGCGCCAAGAAGATCTCCGTCGACA


a3639_g o 95 AGAGCTCCGGCGAGGCCAAGTGCGGCAACGGCCAGAAGCTCGCTTGCTGCAACAGCGGCG
b3639_t o 95 AGAGCTCCGGCGAGGCCAAGTGCGGCAACGGCCAGAAGCTCGCTTGCTGCAACAGCGGCG
c3639_9A 107 AGAGCTCCGGCGAGGCCAAGTGCGGCAACGGCCAGAAGCTCGCTTGCTGCAACAGCGGCG


a3639_g 0155 AGGACCTCATCGGTCTCAACTGCCTGAACGTCCCCGTCCGTACGTATTCCCTCTCGAGCT
b3639_t 0155 AGGACCTCATCGGTCTCAACTGCCTGAACGTCCCCGTCC---------------------
c3639_9A 167 AGGACCTCATCGGTCTCAACTGCCTGAACGTCCCCGTCC---------------------

a3639_g 0215 CCCATCTTCCAAACGGACCTCGAGGCTAACAATCACAACAGTGGCCGTCCCCGTCCAGCA
b3639_t 0194 -----------------------------------------TGGCCGTCCCCGTCCAGCA
c3639_9A 206 -----------------------------------------TGGCCGTCCCCGTCCAGCA


a3639_g 0275 GGCCTGCGGCTCCAACGTCGCCGCGTGCTGCGAGACTGGCGACTCCGAGGGCAACGCCAT
b3639_t 0213 GGCCTGCGGCTCCAACGTCGCCGCGTGCTGCGAGACTGGCGACTCCGAGGGCAACGCCAT
c3639_9A 225 GGCCTGCGGCTCCAACGTCGCCGCGTGCTGCGAGACTGGCGACTCCGAGGGCAACGCCAT


a3639_g 0335 CAACCTCGAGGCCAACTGCGTCTCCATCCCTCTCTAA
b3639_t 0273 CAACCTCGAGGCCAACTGCGTCTCCATCCCTCTCTAA
c3639_9A 285 CAACCTCGAGGCCAACTGCGTCTCCATCCCTCTCTAA

**Fig. S19. Alignment of hydrophobin cDNA sequence (3639_9A) with the predicted DNA sequence (3639_g) and putative coding DNA sequence (3639_t) of *C. sphaerospermum* UM 843.** The start codon is in box and the intron region shaded in yellow. The intron size is 62 bp (position 194-255).

a4115_g 01 ----------------------ATGGCCTTCTTCAAGACTCTCGTCATCGCCTCCGTGG
b4115_t 01 ----------------------ATGGCCTTCTTCAAGACTCTCGTCATCGCCTCCGTGG
c4115_9A 1 AAGCAACTCTCCAACCATCACAATGGCCTTCTTCAAGACTCTCGTCATCGCCTCCGTGG


a4115_g 038 CTGCCGTCGCTGTCGCCCAGGGCGCTTCTGACCACAAGACCGAGATCTCCGCCTCCAAGA
b4115_t 038 CTGCCGTCGCTGTCGCCCAGGGCGCTTCTGACCACAAGACCGAGATCTCCGCCTCCAAGA
c4115_9A 60 CTGCCGTCGCTGTCGCCCAGGGCGCTTCTGACCACAAGACCGAGATCTCCGCCTCCAAGA


a4115_g o 98 GCGAGGACGCTGCCGTCTGCGGCAACGGCCAGAAGATCGCCTGCTGCAACAGCGGCGAGG
b4115_t o 98 GCGAGGACGCTGCCGTCTGCGGCAACGGCCAGAAGATCGCCTGCTGCAACAGCGGCGAGG
c4115_9A 120 GCGAGGACGCTGCCGTCTGCGGCAACGGCCAGAAGATCGCCTGCTGCAACAGCGGCGAGG

a4115_g 0158 ACCTCATCGGTCTTAACTGCCTGAACGTCCCCATCCGTACGTTCCACCATCAATCTATTC
b4115_t 0158 ACCTCATCGGTCTTAACTGCCTGAACGTCCCCATCC------------------------
c4115_9A 180 ACCTCATCGGTCTTAACTGCCTGAACGTCCCCATCC------------------------


a4115_g 0218 GATCGACTAGACCCTTTTAGCTGACAACTCCAAACAGTCGCTGTCCCCATCCAGCAGCGC
b4115_t 0194 -------------------------------------TCGCTGTCCCCATCCAGCAGCGC
c4115_9A 216 -------------------------------------TCGCTGTCCCCATCCAGCAGCGC


a4115_g 0278 TGCGGTTCCAACGTTGCCGCTTGCTGCAAGACTGGCGATGCCGATGTAAGTATCGATCGA
b4115_t 0217 TGCGGTTCCAACGTTGCCGCTTGCTGCAAGACTGGCGATGCCGATG--------------
c4115_9A 239 TGCGGTTCCAACGTTGCCGCTTGCTGCAAGACTGGCGATGCCGATG--------------


a4115_g 0338 TCATTGACAATTCTGACCATCAGCTAACATACTTCTCAGGGCAACCTCATCAACCTCGAG
b4115_t 0263 ----------------------------------------GCAACCTCATCAACCTCGAG
c4115_9A 285 ----------------------------------------GCAACCTCATCAACCTCGAG


a4115_g 0398 GCCAACTGCCTTTCCATCCCGCTTTAA
b4115_t 0283 GCCAACTGCCTTTCCATCCCGCTTTAA
c4115_9A 305 GCCAACTGCCTTTCCATCCCGCTTTAA

**Fig. S20. Alignment of hydrophobin cDNA sequence (4115_9A) with the predicted DNA sequence (4115_g) and putative coding DNA sequence (4115_t) of *C. sphaerospermum* UM 843.** The start codon is in box and the intron region shaded in yellow. The intron size is 61 bp (position 194-254) and 54 bp (position 324-377) respectively.

a6061_g 01 ------------------------ATGGCCTTCTTCAAGACTCTCCTCATCGCCTCCGTG
c6061_t 01 ------------------------ATGGCCTTCTTCAAGACTCTCCTCATCGCCTCCGTG
d6061_9A 1 ----------CCAACACCTTTACAATGGCCTTCTTCAAGACTCTCCTCATCGCCTCCGTG


a6061_g 037 GCTGCCGTCGCCTACGCCGCCCCCCAGGGTGCCGCTGATGGCAAGACCGAGGTCAACGTC
c6061_t 037 GCTGCCGTCGCCTACGCCGCCCCCCAGGGTGCCGCTGATGGCAAGACCGAGGTCAACGTC
d6061_9A 51 GCTGCCGTCGCCTACGCCGCCCCCCAGGGTGCCGCTGATGGCAAGACCGAGGTCAACGTC


a6061_g o 97 AAGTCCGACGACGCCTCCGCCAAGTGCGGCAACGGCCAGAAGCTTGCCTGCTGCAACAGC
c6061_t o 97 AAGTCCGACGACGCCTCCGCCAAGTGCGGCAACGGCCAGAAGCTTGCCTGCTGCAACAGC
d6061_9A 111 AAGTCCGACGACGCCTCCGCCAAGTGCGGCAACGGCCAGAAGCTTGCCTGCTGCAACAGC


a6061_g 0157 GGCGAGGACCTCATCGGCCTGAACTGCCTGAACATCCCCATCCGTGAGTTGACCATTTCC
c6061_t 0157 GGCGAGGACCTCATCGGCCTGAACTGCCTGAACATCCCCATCC-----------------
d6061_9A 171 GGCGAGGACCTCATCGGCCTGAACTGCCTGAACATCCCCATCC-----------------


a6061_g 0217 CATACCACCGAAAGCGCAACGCTGACAGTGCAACAGTTGCTGTCCCCATCCAGCAGACCT
c6061_t 0200 ------------------------------------TTGCTGTCCCCATCCAGCAGACCT
d6061_9A 214 ------------------------------------TTGCTGTCCCCATCCAGCAGACCT


a6061_g 0277 GCGGCTCCAACGTCGCTGCGTGCTGCCAGACTGGCGACGCCTCCGGCAACCTCATCAACC
c6061_t 0224 GCGGCTCCAACGTCGCTGCGTGCTGCCAGACTGGCGACGCCTCCGGCAACCTCATCAACC
d6061_9A 238 GCGGCTCCAACGTCGCTGCGTGCTGCCAGACTGGCGACGCCTCCGGCAACCTCATCAACC


a6061_g 0337 TTGAGGCCAACTGCCTTTCCATCCCGCTTTAA
c6061_t 0284 TTGAGGCCAACTGCCTTTCCATCCCGCTTTAA
d6061_9A 298 TTGAGGCCAACTGCCTTTCCATCCCGCTTTAA

**Fig. S21. Alignment of hydrophobin cDNA sequence (6061_9A) with the predicted DNA sequence (6061_g) and putative coding DNA sequence (6061_t) of *C. sphaerospermum* UM 843.** The start codon is in box and the intron region shaded in yellow. The intron size is 53 bp (position 200-252).

* **

UM843_3639 01 MAFMKTLLIASVAAVAYA---QGGSDGAKKISVDKSSGEAKCGNGQKLACCNSGEDLIGL
UM843_1201 01 MAFIKTLLIASVAALAYAAP-QGASDGNTKVSASKDSQEAACGNGQKLACCNSGEDLIGA
UM843_4115 01 MAFFKTLVIASVAAVAVA---QGASDHKTEISASKSEDAAVCGNGQKIACCNSGEDLIGL
UM843_6061 01 MAFFKTLLIASVAAVAYAAP-QGAADGKTEVNVKSDDASAKCGNGQKLACCNSGEDLIGL
C.herbarum_ClahHCh1 0 1 MAFIKSLLIASVAAVAFAAPQGGASDNNKKVEIDGQDSAPVCGNGQKVACCNSGEDLIGL

 * * ** *
UM843_3639 58 NCLNVPVLAVPVQQACGSNVAACCETGDSEGNAINLEANCVSIPL
UM843_1201 60 NCLNVPILAVPVQQACGSNVAACCKTGDASGNLINLELNCLSLPL
UM843_4115 58 NCLNVPILAVPIQQRCGSNVAACCKTGDADGNLINLEANCLSIPL
UM843_6061 60 NCLNIPILAVPIQQTCGSNVAACCQTGDASGNLINLEANCLSIPL
C.herbarum_ClahHCh1 061 NCLSIPILAIPIQKACGSNIAACCQTGDSEGNLLNLEANCLAIPL

**Fig. S22. Multiple sequences alignment of putative hydrophobin genes (UM843_3639, UM843_1201, UM843_4115, UM843_6061) with hydrophobin gene from *Cladosporium herbarum* Cla HCh 1 (Q8NIN9).** The eight conserved cysteine (C) residues were marked in asterisk (*).

References:

1. Zhao, Z., Liu, H., Wang, C. & Xu, J.-R. Correction: Comparative analysis of fungal genomes reveals different plant cell wall degrading capacity in fungi. *BMC Genomics* **15,** 6 (2014).

2. Ohm, R. A. *et al.* Diverse lifestyles and strategies of plant pathogenesis encoded in the genomes of eighteen Dothideomycetes fungi. *PLoS Pathog.* **8,** e1003037 (2012).

3. Islam, M. S. *et al.* Tools to kill: genome of one of the most destructive plant pathogenic fungi *Macrophomina phaseolina*. *BMC Genomics* **13,** 493 (2012).
